# Role of human Pegivirus infections in whole *Plasmodium falciparum* sporozoite vaccination and controlled human malaria infection in African volunteers

**DOI:** 10.1186/s12985-021-01500-8

**Published:** 2021-01-26

**Authors:** Anneth-Mwasi Tumbo, Tobias Schindler, Jean-Pierre Dangy, Nina Orlova-Fink, Jose Raso Bieri, Maximillian Mpina, Florence A. Milando, Omar Juma, Ali Hamad, Elizabeth Nyakarungu, Mwajuma Chemba, Ali Mtoro, Kamaka Ramadhan, Ally Olotu, Damas Makweba, Stephen Mgaya, Kenneth Stuart, Matthieu Perreau, Jack T. Stapleton, Said Jongo, Stephen L. Hoffman, Marcel Tanner, Salim Abdulla, Claudia Daubenberger

**Affiliations:** 1 Department of Intervention and Clinical Trials, Ifakara Health Institute, Bagamoyo, Tanzania; 2 Department of Medical Parasitology and Infection Biology, Clinical Immunology Unit, Swiss Tropical and Public Health Institute, Socinstr. 57, 4002 Basel, Switzerland; 3 University of Basel, Basel, Switzerland; 4 Equatorial Guinea Malaria Vaccine Initiative, Malabo, Bioko Norte Equatorial Guinea; 5 Center for Global Infectious Disease Research, Seattle Children’s Research Institute, 307 Westlake Avenue, N. Suite 500, Seattle, WA 98109 USA; 6 Centre Hospitalier Universitaire Vaudois, Lausanne, Switzerland; 7 Iowa City Veterans Administration and the University of Iowa, 200 Hawkins Drive, Iowa City, IA 52242 USA; 8 Sanaria Inc, Rockville, MD 20850 USA; 9 Dar-Es-Salaam Institute of Technology, Dar-Es-Salaam, Tanzania; 10 Tanzania Education and Research Networks, Dar-Es-Salaam, Tanzania; 11 Tanzania Commission for Science and Technology, Dar-Es-Salaam, Tanzania

**Keywords:** Malaria, Human pegivirus, Controlled human malaria infection, Immune activation, Antibody response, PfSPZ vaccine

## Abstract

**Background:**

Diverse vaccination outcomes and protection levels among different populations pose a serious challenge to the development of an effective malaria vaccine. Co-infections are among many factors associated with immune dysfunction and sub-optimal vaccination outcomes. Chronic, asymptomatic viral infections can contribute to the modulation of vaccine efficacy through various mechanisms. Human Pegivirus-1 (HPgV-1) persists in immune cells thereby potentially modulating immune responses. We investigated whether Pegivirus infection influences vaccine-induced responses and protection in African volunteers undergoing whole *P. falciparum* sporozoites-based malaria vaccination and controlled human malaria infections (CHMI).

**Methods:**

HPgV-1 prevalence was quantified by RT-qPCR in plasma samples of 96 individuals before, post vaccination with PfSPZ Vaccine and after CHMI in cohorts from Tanzania and Equatorial Guinea. The impact of HPgV-1 infection was evaluated on (1) systemic cytokine and chemokine levels measured by Luminex, (2) PfCSP-specific antibody titers quantified by ELISA, (3) asexual blood-stage parasitemia pre-patent periods and parasite multiplication rates, (4) HPgV-1 RNA levels upon asexual blood-stage parasitemia induced by CHMI.

**Results:**

The prevalence of HPgV-1 was 29.2% (28/96) and sequence analysis of the 5′ UTR and E2 regions revealed the predominance of genotypes 1, 2 and 5. HPgV-1 infection was associated with elevated systemic levels of IL-2 and IL-17A. Comparable vaccine-induced anti-PfCSP antibody titers, asexual blood-stage multiplication rates and pre-patent periods were observed in HPgV-1 positive and negative individuals. However, a tendency for higher protection levels was detected in the HPgV-1 positive group (62.5%) compared to the negative one (51.6%) following CHMI. HPgV-1 viremia levels were not significantly altered after CHMI.

**Conclusions:**

HPgV-1 infection did not alter PfSPZ Vaccine elicited levels of PfCSP-specific antibody responses and parasite multiplication rates. Ongoing HPgV-1 infection appears to improve to some degree protection against CHMI in PfSPZ-vaccinated individuals. This is likely through modulation of immune system activation and systemic cytokines as higher levels of IL-2 and IL17A were observed in HPgV-1 infected individuals. CHMI is safe and well tolerated in HPgV-1 infected individuals. Identification of cell types and mechanisms of both silent and productive infection in individuals will help to unravel the biology of this widely present but largely under-researched virus.

## Background

Vaccination is an invaluable tool in public health that has contributed to control of many, and in some cases, to the elimination of infectious disease like smallpox [1]. Malaria, a disease caused by *Plasmodium* species remains a major public health burden particularly in the tropics and sub-tropical regions where it accounted for approximately 405,000 deaths in 2018 [2]. A major goal in malaria research is to develop an efficacious vaccine that complements currently used control tools based on vector control and treatment of clinical malaria infections [3]. However, these vaccine development efforts are challenged by an incomplete understanding of the immune mediators leading to highly protective, long-lasting vaccine induced immunity in the field [4]. A number of studies testing cryopreserved, purified, metabolically active and radiation-attenuated whole sporozoites of *P. falciparum* as vaccine approach (PfSPZ Vaccine) have been published recently [5–9]. Strikingly, the comparison of PfSPZ vaccine-induced antibody titers specific for the *P. falciparum* circumsporozoite protein (PfCSP) showed significantly lower titers in malaria pre-exposed than malaria-naive individuals immunized with the PfSPZ Vaccine using comparable regimen [6–9]. These differences in PfSPZ vaccine-induced immunity was also observed between vaccinees residing in malaria endemic countries including Tanzania, Mali and Equatorial Guinea [10–12].

Recently our group demonstrated that age, location and iron status influence the immune system development of children as well as vaccine-induced responses to the most advanced malaria vaccine candidate, the RTS,S [13]. Additionally, communicable and non-communicable diseases have been implicated in suboptimal vaccine-induced responses [14] Chronic, asymptomatic viral infections at time of immunization might contribute to reduced magnitude and longevity of vaccine-induced immune responses [15–17]. To date, the number of human viruses investigated in this context is limited and their mechanisms in modulation of vaccine-induced responses remain unclear.

Human Pegivirus-1 (HPgV-1), a predominantly asymptomatic virus causing a chronic infection, is common in Africa where an estimated 18–28% of its roughly 750 million global infections occur [18]. The virus establishes its persistence potentially by replicating in immune cells including T cells, B cells, monocytes, and natural killer (NK) cells [19, 20]. Interestingly, seminal field studies have linked HPgV-1 co-infection status to significant survival advantages in HIV-1 and Ebola infected humans [21–24]. These favourable outcomes are thought to be based on immune-modulatory properties of HPgV-1 such as reduced activation of T cells, B cells and NK cells [20, 25] and the altered regulation of cytokine and chemokine expression [26–28]. Different HPgV-1 genotypes might influence the extent of immune modulation resulting in varied disease outcomes [21–23].

Given the high prevalence of HPgV-1 infection in *P. falciparum* endemic countries, we expected an overlapping geographical distribution and aimed to investigate within-host interactions between the two infections. We were therefore interested to study whether HPgV-1 infection status might influence PfSPZ vaccine-induced immune responses. We characterized prevalence and genotype distribution of HPgV-1 in three cohorts of adult volunteers participating in PfSPZ Vaccine studies [9, 29, 30]. We explored the influence of HPgV-1 infection status on cytokine and chemokine levels in serum and correlated HPgV-1 infection on vaccine-induced anti-PfCSP-antibody titers and protection against homologous CHMI. We also aimed to characterize for the first time the potential impact of a CHMI study on HPgV-1 viremia in these volunteers.

## Methods

### Study population

We used samples from volunteers enrolled in four studies conducted in Bagamoyo, Tanzania (acronyms BSPZV1, BSPZV2, and BSPZV3a) and on Bioko Island in Equatorial Guinea (acronym EGSPZV2) registered at Clinicaltrials.gov with registration numbers NCT03420053, NCT02132299, NCT02613520, and NCT02859350, respectively. Detailed trial designs and study procedures such as pre-defined inclusion and exclusion criteria have been described previously [9, 10, 29, 30]. Briefly, these trials evaluated the safety, immunogenicity and efficacy of live, cryopreserved, purified, whole *P. falciparum* sporozoites in malaria pre-exposed volunteers. The analyses in these studies were performed on samples collected from adult volunteers in which vaccine efficacy was evaluated by homologous CHMI based on direct intravenous inoculation of 3200 fully infectious, aseptic purified cryopreserved *P. falciparum* sporozoites. The current study was performed in two parts (i) a pilot virome study that included samples from a subset of volunteers from BSPZV1 (NCT03420053) (Additional file 1: Fig. 1); (ii) the main study which utilized samples from volunteers participating in the BSPZV2 (NCT02132299), BSPZV3a (NCT02613520) and EGSPZV2 (NCT02859350) trials. Samples were selected based on availability and scientific aims. Tested sample types and sizes are described in further detail in each section and in Additional file 1: Fig. 1A.

### Identification of human Pegivirus RNA in RNA-seq data from whole blood

Whole venous blood samples were used from a subset of participants (n = 28) (Additional file 1 Fig. 1A) participating in the BSPZV1 vaccine trial (NCT03420053) based on their availability. All volunteers were healthy males, aged 18 to 35 years and confirmed as negative for HIV-1, Hepatitis B and C at enrolment. Venous blood was collected at different time points including: before vaccination (baseline), 2 and 7 days after first vaccination, 7 days after the second vaccination, before CHMI, 2 and 9 days after CHMI. Each of the placebo (n = 6) and the vaccine (n = 22) participants had a total of 3 and 7 blood sampling time points screened respectively, resulting in 172 samples in total. All blood samples (n = 172) were stored in Paxgene RNA tubes and subjected to RNA-seq analysis performed as published [31]. Briefly, RNA-seq data was generated from whole blood RNA (depleted for globin/rRNA) that was fragmented; the first strand cDNA synthesis was done by random priming and dTTP was used, whereas 2nd strand cDNA synthesis used dUTP which eliminates 2nd strands in the downstream PCR amplification that enabled strand specific RNA-seq sequencing [31]. From the RNA-seq sample set (n = 172), 800 million non-human reads were identified. Given the naturally occurring fluctuation of viremia of many viruses, we performed a longitudinal assessment of viral infection status, and obtained reads from all available time points for each of the volunteers. The analyses was performed in an in-house developed viral metagenomics analysis pipeline outlined in (Additional file 1: Fig. 1B–C). The pipeline is a combination of several published algorithms adapted from commonly used viral metagenomic analytical tools [32–36]. Briefly, our analyses were carried out in three consecutive steps: viral identification, in silico validation and RT-PCR confirmation. In the viral identification step, we analysed approximately 3 million non-human, unmapped paired end reads from each volunteer. The initial reads were first searched for “suspected” viral hits by running bowtie2 against the NCBI database containing more than 7424 viral genomes. Thereafter, low quality and complexity reads as well as reads mapping to human genome, transcriptome and repeat regions were removed from the resulting “suspected” viral reads using bowtie2, knead data and tandem repeat finder algorithms, respectively. The “clean” viral reads were then comprehensively searched for viral hits using virome scan [32] and Taxonomer [33] and for viral proteins using adapted Diamond tool containing a custom made database with more than 100,000 viral proteins [35]. The initial unmapped reads were also analysed by Fast virome explorer without filtering for host reads to allow the identification of endogenous retroviral elements and other viruses that may have been missed previously [34]. Only viral hits known to be associated with a human host were selected, and viral contaminants such as lymphotropic murine virus and synthetic constructs coding for either HIV-1 or hepatitis B were removed based on documented literature [37]. In a following *in-silico* confirmatory step, the suspected viral hits were blasted and mapped against specific viral whole genomes using a Geneious bioinformatics tool [38]. As a last step, we performed reverse transcription PCR (RT-PCR) analysis. Due to limited sample availability we were unable to screen the entire BSPZV1 cohort for HPgV-1. Hence, the presence of the most prevalent virus (human pegivirus-1, HPgV-1) was confirmed by RT-PCR in plasma samples of volunteers found positive by RNA-seq transcriptome analysis only.

**Fig. 1:**
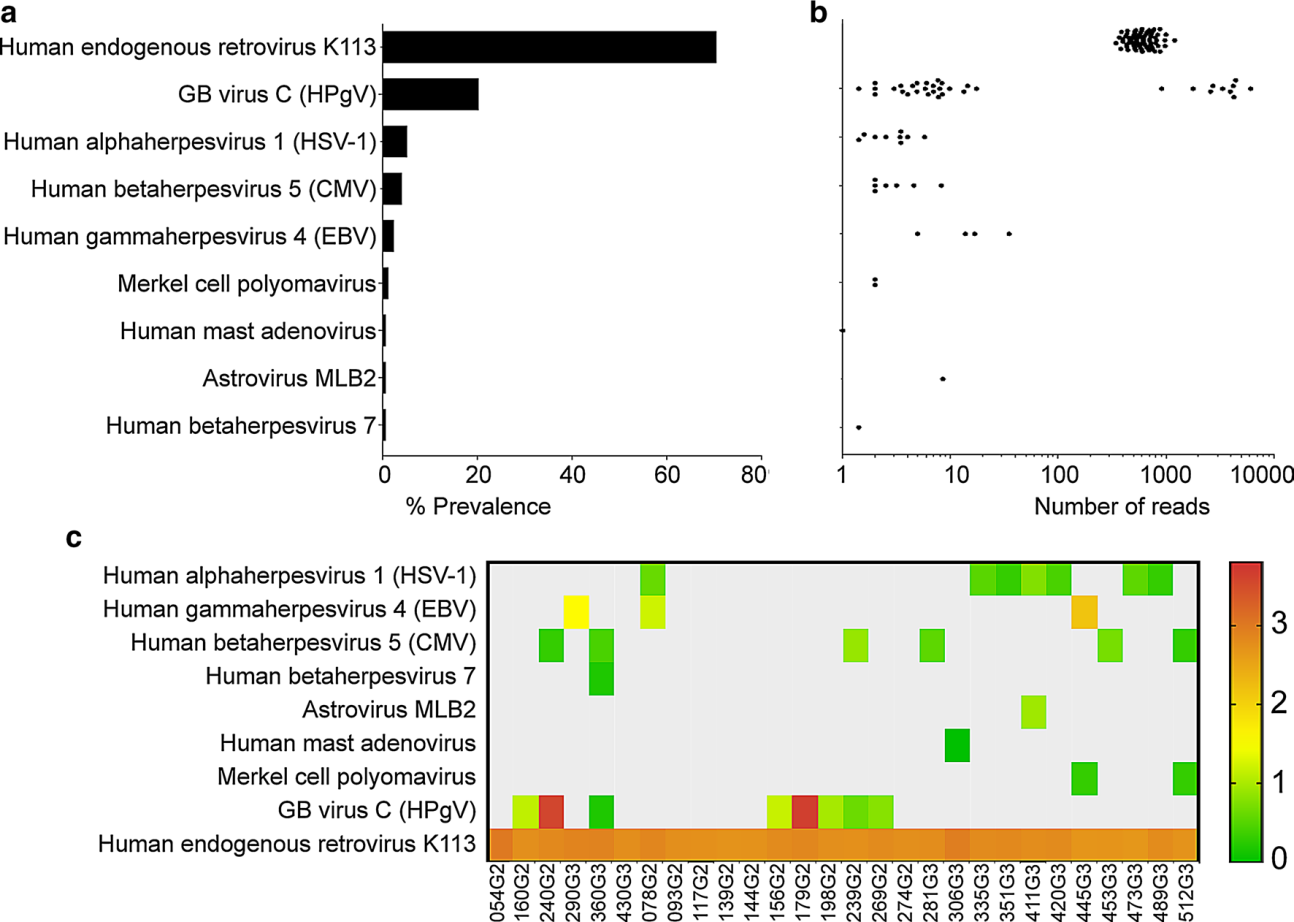
Unbiased search for RNA molecules encoding human viruses in RNA-seq transcriptomics data. **a** Overall prevalence of 9 human viruses detected in 172 whole blood samples **b** Number of viral RNA-seq reads detected for each of the identified viruses. Virus names are plotted on the y-axis and proportion (**a**), number of reads (**B**) on the x-axis. **c** Distribution of the 9 different viruses across the 28 individuals included. Virus names are plotted on the y-axis and volunteer IDs on the x-axis. Each bar indicates viral reads for an individual. The log viral RNA-seq reads are plotted, in increasing order ranging from 0 to 3; green indicating low number and red high number of reads

### RT-qPCR for detection and quantification of HPgV-1 and HPgV-2

Plasma samples collected from male and female individuals (n = 96), aged 18–45 years, and participating in the BSPZV2, BSPZV3a and the EGSPZV2 studies were included. Plasma was prepared by density gradient centrifugation of whole blood and cryopreserved. At analysis cryopreserved plasma was thawed and used for detection of HPgV-1 and HPgV-2 RNA in all study participants. Plasma samples collected at 3 time points for each volunteer were included, namely before vaccination (baseline), before CHMI and 28 days after CHMI. Presence or absence of HPgV-1 and HPgV-2 was determined simultaneously using RT-qPCR based on published methods [25]. Briefly, total nucleic acids were extracted from 300 ul plasma using Zymo quick DNA/RNA viral kit (Zymo Research, Irvine, USA) and eluted in 50 ul of DNase/RNase free water. 5 ul of the recovered DNA/RNA solution was used as amplification template together with 2X.Lunar universal one step qPCR master mix (10 ul, 1X), Luna warm start reverse transcriptase enzyme mix (1 ul, 1X) (New England Biolabs, MA, USA) and primers binding to the 5′ untranslated regions of HPgV-1 and HPgV-2 (each at 2 ul, 0.4 uM) [25, 39]. In addition, human RNaseP primers were added as internal control. Each sample was run in triplicate in a one-step multiplex RT-qPCR using the CFX96 real time PCR system (Bio-Rad, Hercules, CA, USA). The RT-qPCR cycling conditions were: 55 °C for 10 min, 95 °C for 1 min, 45 cycles at 95 °C for 15 s and 55 °C for 1 min. The generated data were uploaded to an in-house available analysis platform where quantification cycle values (Cq) were calculated automatically [40]. HPgV viral quantification was done as described by Stapleton et al. using in vitro transcribed (IVT) viral RNA [25]. In each RT-qPCR experiment, we included a positive (HPgV-1 IVT-RNA), a negative (from HPgV-1 negative volunteer) and a non-template control.

### Genotyping of HPgV-1

The Fire Script cDNA kit was used to synthesize cDNA in accordance to manufacture instructions (Solis Biodyne, Tartu, Estonia). Briefly, 5 ul of extracted RNA as described above was added into a master mix containing forward and reverse primers specific to 5′ UTR of HPgV-1 (each at 1,1 uM**)**, deoxribonucleotide triphosphate mix (dNTP) (0.5 ul, 500 uM**)**, reverse transcription buffer with DTT (2 ul, X1), RiboGrip Rnase inhibitor (0.5 ul, 1 U/ul), Fire script reverse transcriptase (Solis Biodyne, Tartu, Estonia) (2 ul, 10 U/ul) and RNase free water (9 ul to 20 ul). Amplification conditions included 50 min at 50 °C and 10 min at 94 °C. 3 ul of cDNA generated by reverse transcription were used for the first round of PCR amplification with forward primer 5′-AAAGGTGGTGGATGGGTGATG-3′ [41] and reverse primer 5′-ATG CCACCCGCCCTCACCAGAA-3′ combination [41]. 1.2 ul of this amplification product was then used for the nested PCR amplification using the forward primer 5′-AATCCC GGTCAYAYTGGTAGCCACT-3′ and reverse primer 5′-CCCCACTGGCZTTGYCAACT-3′ combination [41]. Both PCR reactions included primers specific for HPgV-1 (1 ul, 1 uM**)**, firepol master mix (4 ul, X5) **(**Solis Biodyne, Tartu, Estonia**)** and RNase free water to a final volume of 20 ul. Cycling conditions were 5 min at 95 °C, followed by 28 cycles of 95 °C for 30 s, 56 °C for 30 s and 72 oC for 30 s with a final extension step at 72 °C for 10 min. The E2 region was amplified as described by Souza et al. [42]. The final PCR products from 5′ UTR amplification (256 base pairs) and E2 amplification (347 base pairs) were sequenced by the Sanger sequencing method (Microsynth, Switzerland).

### HPgV-1 phylogenetic analysis

Nucleotide sequence analysis and phylogenetic analysis was performed with the Geneious software version 8.1.9. Chromatograms were examined for quality, and only sequences with quality threshold above 86% were included in analysis. CLUSTALW algorithm was used to align 5` UTR nucleotide sequences from volunteers to selected reference sequences corresponding to 5` UTR of HPgV-1 (genotype 1 to 7) available through the NCBI database. Thereafter, phylogenetic trees were constructed by the neighbour joining method and the Kimura two parameter models. The references sequences for 5` UTR of HPgV-1 included AF488786, AF488789, KC618399, KP710602, U36388, JX494177, Y16436, and MF398547 (Genotype 1); AB003289, AF104403, D90600, JX494179, MG229668, JX494180, U4402, U59518 (Genotype 2; 2a), MH000566, U59529, U63715, MH053130 (Genotype 2; 2b); AB008335, KR108695, JX494176, D87714 (Genotype 3); AB0188667, AB021287, HQ3311721 (Genotype 4); DQ117844, AY949771, AF488796, AF488797 (Genotype 5); AB003292, AF177619 (Genotype 6), HQ331235, HQ 3312233 (Genotype 7). The hepatitis C nucleotide sequence deposited under AJ132997 was used as an out-group. For the E2 region the sequences were KP701602.1, KM670109, U36380, KP710600, KC618399, AB003291 (Genotype 1); AF121950, MK686596, D90600 (Genotype 2a) and U63715 (Genotype 2b); D87714 (Genotype 3); AB0188667 (Genotype 4); AY949771, KC618401, AY951979 (Genotype 5); AB003292 (Genotype 6). A Chimpanzee HPgV-1 strain deposited under AF70476 was used as an out-group and U4402 (Genotype 2) was used for mapping of our sequences to identify regions of similarity.

### Ex vivo* cytokine and chemokine measurements*

Serum samples available from 44 volunteers collected from EGSPZV2 and BSPZV3a (only HIV-1 negative volunteers) at baseline were used for the assessment of the systemic immune activation status. Cytokine and chemokine concentrations were measured using the Cytokine/Chemokine/Growth Factor 45-Plex Human ProcartaPlex™ Panel 1 (Affymetrix Biosciences, USA) and acquired on a validated Luminex XMAP technology platform as described [43]. The investigated cytokines and chemokines included BDNF, Eotaxin/CCL11, EGF, FGF-2, GM-CSF, GRO alpha/CXCL1, HGF, NGF beta, LIF, IFN alpha, IFN gamma, IL-1 beta, IL-1 alpha, IL-1RA, IL-2, IL-4, IL-5,IL-6, IL-7, IL-8/CXCL8, IL-9, IL-10, IL-12 p70, IL-13, IL-15, IL-17A, IL-18, IL-21, IL-22, IL-23, IL-27, IL-31, IP-10/CXCL10, MCP-1/CCL2, MIP-1 alpha/CCL3, MIP-1 beta/CCL4, RANTES/CCL5, SDF-1 alpha/CXCL12, TNF alpha, TNF beta/LTA, PDGF-BB, PLGF, SCF, VEGF-A and VEGF-D. Only cytokines and chemokines with levels above the pre-defined lower detection limit of the specific standard curves were included in the group comparisons. Absolute concentrations were normalized to account for the inter-plate variations before analysis in R software version 3.5.1.

### Serological analysis

Serum samples for anti-PfCSP antibody evaluation were collected before vaccination (baseline) and 14 days post last vaccination. Anti-PfCSP total IgG levels were measured by enzyme linked immunosorbent assay (ELISA) as described previously [9, 10, 29, 30].

### Quantitative detection of Plasmodium falciparum

Asexual blood-stage malaria parasitemia during CHMI was assessed using thick blood smear (TBS) microscopy and retrospectively analysed using stored whole blood samples and qPCR as described [9, 10, 29, 30]. Whole blood samples for the assessment of parasitemia were taken before CHMI and during the observation period beginning at day 9 after parasite challenge inoculation until volunteers either became asexual blood-stage malaria positive or until day 21. TBS were performed twice a day from day 9 to 14 and then once a day for day 15 to 21. TBS were also performed on day 28 before malaria drug treatment. Pre-patent periods were calculated from the time of PfSPZ challenge to first positivity detected by qPCR and TBS [9, 10, 29, 30]. Parasite multiplication rate (PMR) was assessed using a linear model fitted to log10-transformed qPCR data as published [44]. PMR was calculated for all volunteers that developed asexual blood-stage parasitemia which lasted for at least two 48-h cycles [44].

### Statistical analysis

Figures and statistical analyses were generated in R version 3.5.1 and GraphPad Software (Prism V5). Wilcoxon rank sum test or Mann–Whitney test were used to compare continuous variables. Chi-square test was used to compare categorical variables. Absolute values for antibody titers and concentrations of cytokines and chemokines were plotted. Data were log transformed only when investigating the anti-PfCSP antibody titres and viremia levels. Spearman correlation was used to investigate the potential effect of HPgV-1 infection status and viremia with antibody titres and cytokine levels. Data for cytokines, chemokines and growth factors were not analysed for multiple correction as we considered this question as exploratory. *P *value ≤ 0.05 was considered significant. Differences in viral diversity, abundance and prevalence were assessed using Linear discriminant analysis effect size [45] and GraphPad Software (Prism V5), respectively.

## Results

### Unbiased search for RNA molecules encoding human viruses

We aimed to identify viruses present in peripheral blood of our volunteers participating in PfSPZ Vaccine studies. These analyses included samples from 28 participants of the BSPZV1 study collected at multiple time points including baseline, 2 days after first vaccination, 7 days after the first and second vaccination and before CHMI, 2 and 9 days after CHMI. Sequences were identified from a pool of RNA-seq data reads that did not map to the human reference transcriptome. A total of 800 million non-human RNA-seq reads derived from 172 whole blood samples were analysed with our virome discovery platform based on previously established metagenomics pipelines and tools (Additional file 1: Fig. 1B, C) [32–35].

In total, RNA molecules encoding 9 human viruses were detectable including the Human simplex virus (HSV-1), Cytomegalovirus (CMV), Epstein-Barr virus (EBV), Merkel cell polyomavirus (MCV), Human mast adenovirus (HAdV), Astrovirus MBL2, Human betaherpesvirus 7 (HHV-7), Human endogenous retrovirus K113 (HERV-K113) and HPgV-1 (Fig. 1a). The number of reads for each of the identified viruses was quantified and is given in Fig. 1b. After identifying 9 different viruses present in 172 whole blood samples, we further assessed the distribution of viruses within our cohort. HERV-K113 was detected with high number of reads in all 28 individuals, while HSV-1 and CMV were present in seven and six individuals, respectively (Fig. 1c). MBL2, HHV-7 and HAdV were present in low read counts in one individual each, and MCV was found in two individuals. Eight individuals carried HPgV RNA with read counts ranging from low to high (Fig. 1c). Three out of 8 HPgV-positive individuals were co-infected with CMV (Fig. 1c). Our analysis showed that a high proportion of Tanzanian adults (28.6%, 8/28) harboured HPgV -1 infection. To reconfirm our findings, we extracted RNA from plasma samples collected from these 8 volunteers and amplified HPgV-1 by RT-PCR. We reconfirmed in 2 out of 8 volunteers the in silico identified presence of HPgV-1 RNA. Interestingly, these 2 volunteers had the highest RNA read counts for HPgV-1 in our bioinformatics analysis (Fig. 1c). These results were important for selecting HPgV-1 as our further research focus and the optimization of RT-PCR assay used for assessment of HPgV-1 infection status in the main study described below.

### Prevalence of HPgV-1 in East and West African volunteers

**Fig. 2:**
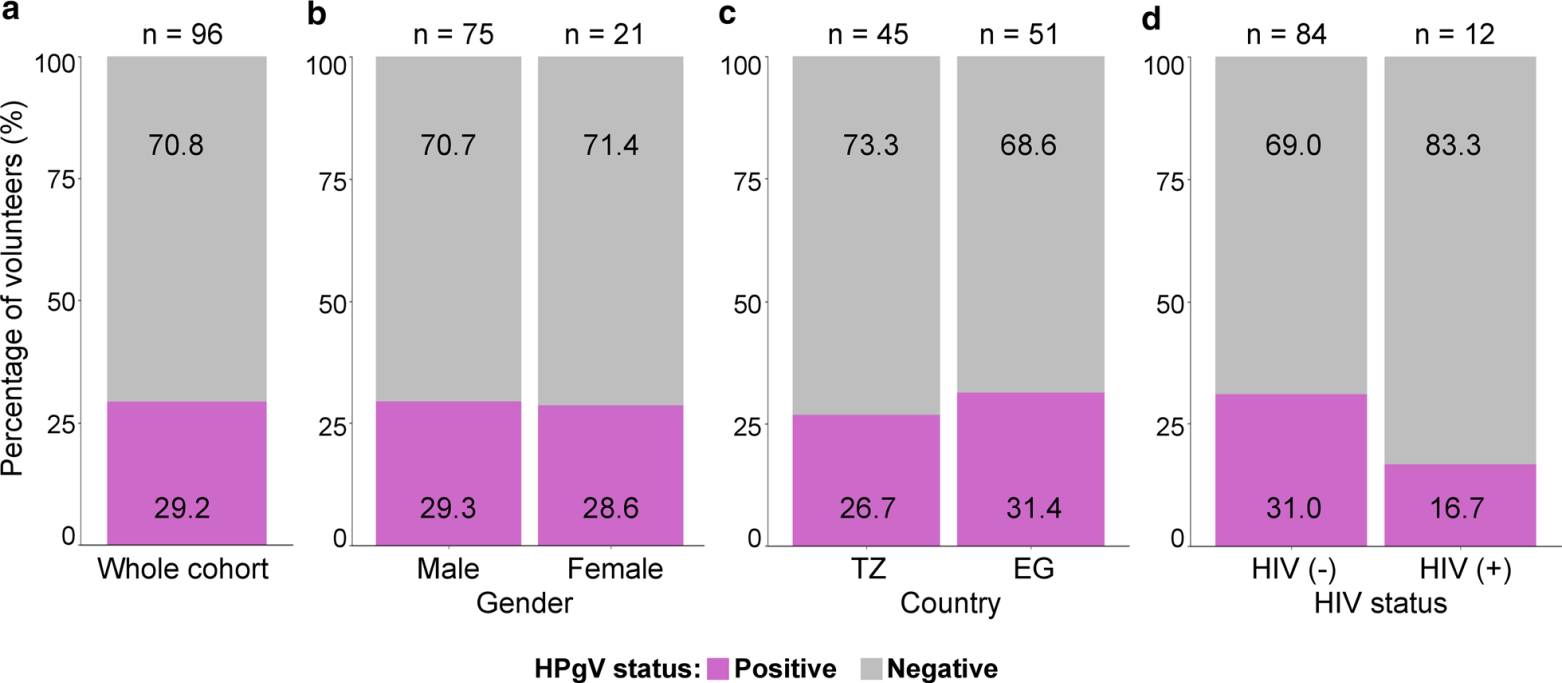
Proportion of individuals with (purple) and without (grey) HPgV- 1 infection. **a** Total cohort of 96 vacinees, **b** separated by gender, **c** Country of origin, **d** HIV-1 infection status. All individuals are between 18 and 35 years of age. Chi square with Yates correction for group comparisons (*P < 0.05)

After having established that HPgV-1 is highly present in our Tanzanian cohort (BSPZV1), we aimed to explore the prevalence of HPgV-1 and HPgV-2 in two larger cohorts from Tanzania (BSPZV2 and BSPZV3a) and one from Equatorial Guinea (EGSPZV2). Plasma samples collected from 96 participants, including 12 HIV-1 positive individuals, were analysed for presence of HPgV-1 and HPgV-2 using optimized RT-qPCR. The overall prevalence of HPgV-1 was 29.2% (28/96) (Fig. 2a), while HPgV-2 was not detected. The proportion of HPgV-1 positive individuals by gender and geographic location were comparable, with slightly more HPgV-1-positive individuals in Equatorial Guinea (31.4%) than Tanzania (26.7%) (Fig. 2b, c). Of the 12 HIV-1 positive individuals from the BSPZV3a study, two (16.7%, 2/12) were positive for HPgV-1 (Fig. 2d).

### HPgV-1 viral loads and distribution

**Fig. 3:**
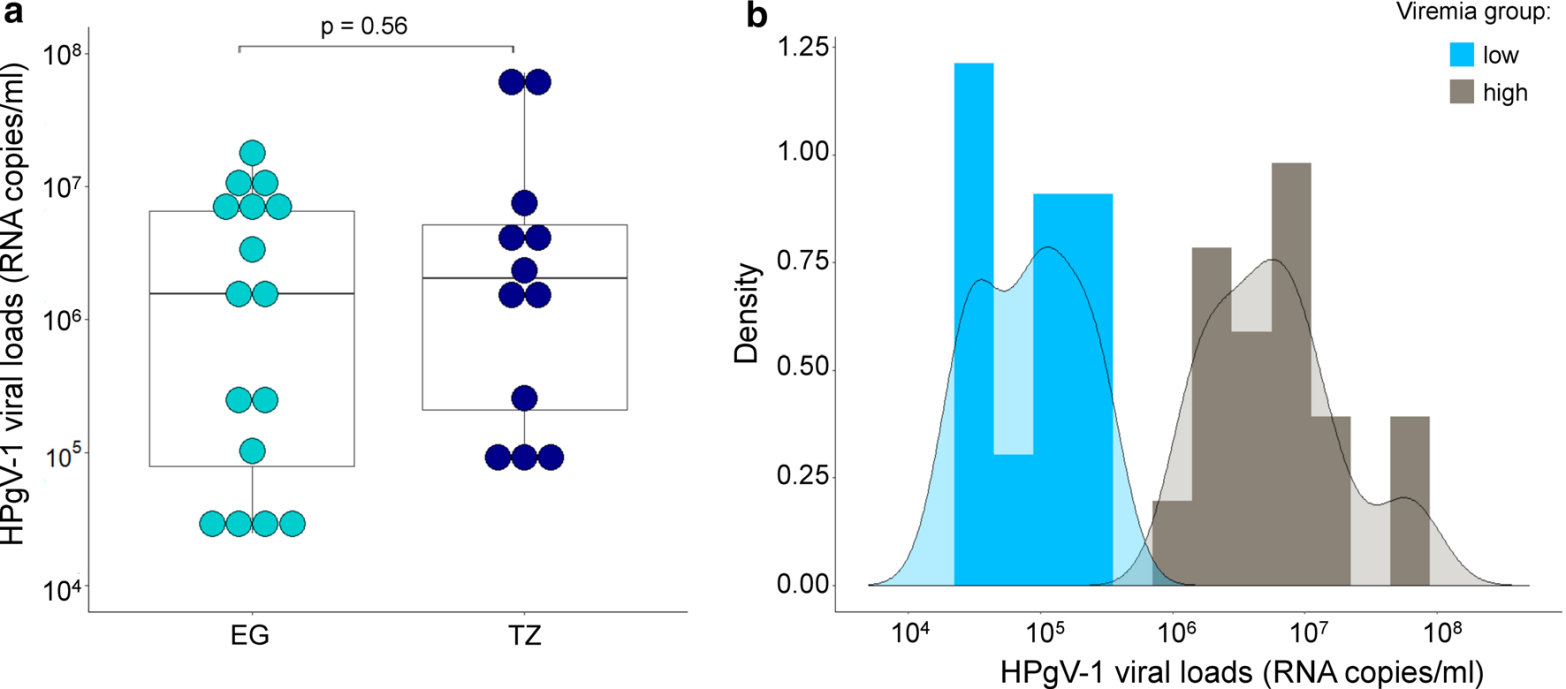
Comparisons of HPgV-1 viral loads. No differences in HPgV-1 viral loads between Equatorial Guinea (green, n = 16**)** and Tanzania (blue, n = 12) volunteers **(a)**. Two distinct groups with low (blue) and high (grey) viremia levels in plasma are found in HPgV-1 infected individuals **(b).** The two groups were divided based on a cut off value of 600,000 RNA copies/ml plasma

Next, we quantified the HPgV-1 viral load in plasma samples using RT-qPCR. HPgV-1 viral loads were comparable between individuals from the two countries (Fig. 3a). However, based on viral loads with a predefined threshold of 10^6^ viral RNA copies/ml of plasma, both cohorts could be divided into HPgV-1 low and high viremic individuals (Fig. 3b). High and low HPgV-1 viremia were found in 17 (60%) and 11 (40%) of the 28 HPgV-1 positive volunteers, respectively (Fig. 3b). Of the 17 high viremic individuals, 8 were from Tanzania and 9 from Equatorial Guinea. Of the 11 low viremic individuals, 4 were Tanzanians and 7 Equatorial Guineans.

### Genotyping of HPgV-1 isolates

**Fig. 4:**
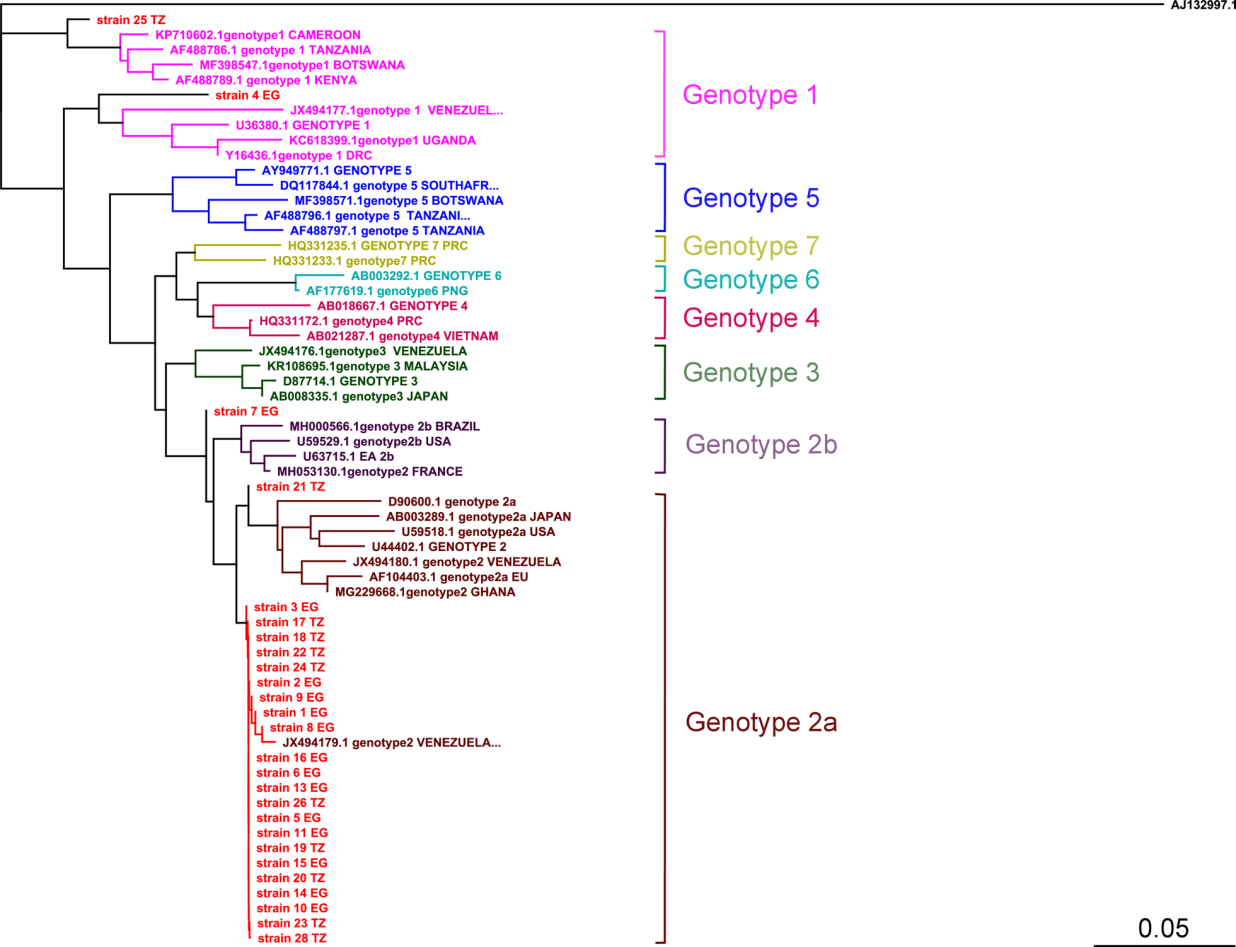
Phylogenetic inferences of the HPgV-1 isolates based on 5′ UTR. Phylogenetic tree was constructed using Neighbour joining method and Kimura two-parameter model of the 5′ UTR. The 5′ UTR sequences from Tanzania and Equatorial Guinea (n = 26) were compared to selected references spanning genotype 1 to 7 from different countries available in the NCBI database. The accession numbers for the reference sequences were: AF488786, AF488789, KC618399, KP710602, U36388, JX494177, Y16436, and MF398547 (Genotype 1, Pink); AB003289, AF104403, D90600, JX494179, MG229668, JX494180, U4402, U59518 (Genotype 2; 2a light brown), MH000566, U59529, U63715, MH053130 (Genotype 2; 2b Brown); AB008335, KR108695, JX494176, D87714 (Genotype 3, Green); AB0188667, AB021287, HQ3311721 (Genotype 4, Maroon); DQ117844, AY949771, AF488796, AF488797 (Genotype 5, Light blue), AB003292, AF177619 (Genotype 6, Bright green); HQ331235, HQ 3312233 (Genotype 7, Golden) and Hepatitis C (AJ132997, Black) was used as outgroup

**Fig. 5:**
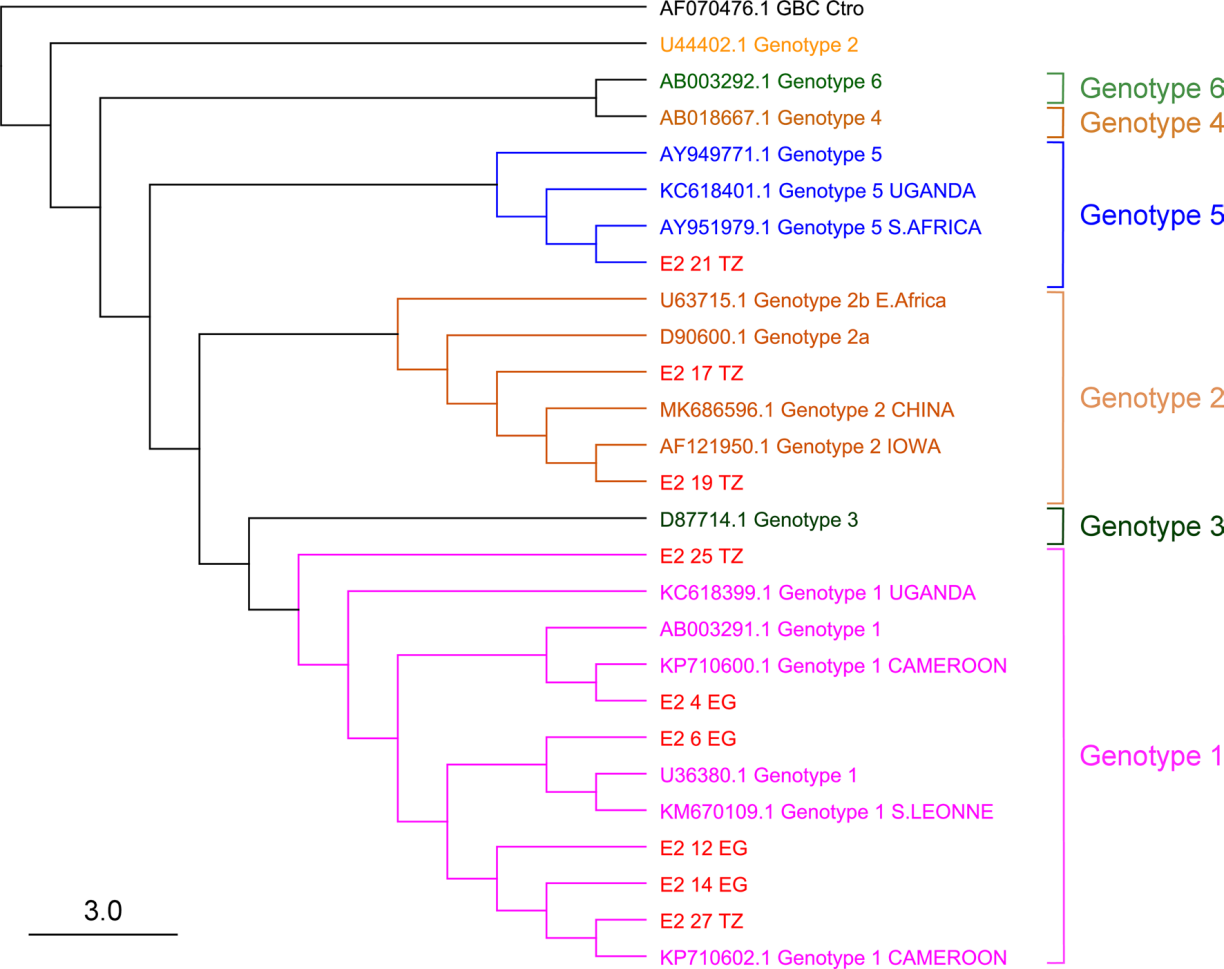
Phylogenetic inferences of the HPgV-1 isolates based on E2 region. Phylogenetic tree was constructed using Neighbour joining method and Kimura two-parameter model of the E2 region of HPgV-1. The E2 sequences from Tanzania and Equatorial Guinea (n = 9) were compared to selected references spanning genotype 1 to 6 from different countries available in the NCBI database including; KP701602.1, KM670109, U36380, KP710600, KC618399, AB003291 (Genotype 1, Pink); AF121950, MK686596, D90600 (Genotype 2; 2a Brown), U63715 (Genotype 2; 2b Brown) D87714 (Genotype 3, Green); AB0188667 (Genotype 4, Brown); AY94977, KC618401, AY951979 (Genotype 5, Light blue) and AB003292 (Genotype 6, Green). Equatorial Guinean and Tanzanian strains identified in this study are denoted by strain number followed with letters EG or TZ, respectively (Red). Chimpanzee HPgV-1 strain (AF70476, Black) was used as outgroup and U4402 (Genotype 2, Golden) was used for mapping of our sequences to identify regions of similarity. The scale bar under the tree indicates nucleotide substitution per site

Seven different genotypes of HPgV-1 have been described globally with genotype 1 and 5 being highly prevalent in sub-Saharan Africa [26]. Therefore, we determined the phylogenetic relatedness of the isolates by amplifying and sequencing the 5′ UTRs. From the 28 positive individuals, 2 were excluded due to poor quality of the sequences obtained. Genotype 1 was found in 2 volunteers (7.7%) and surprisingly, genotype 2, described as dominant in Europe and America, was found in 24 of 26 volunteers (92.3%) (Fig. 4). Most genotype 2 strains clustered closely with the related genotype 2a sequences described from Venezuela (Fig. 4). To further increase the resolution of the genetic relatedness of our isolates, we amplified in addition the polymorphic E2 region of HPgV-1. E2 RNA was successfully amplified and sequenced in 9 of the 28 positive volunteers (32%). According to the E2-sequences of our HPgV-1 isolates, our strains clustered within genotypes 1, 2, and 5 (Fig. 5). Notably, genotypic clustering of isolates 6EG and 14EG differed based on E2 and 5′ UTR derived PCR products (Figs. 4, 5). In summary, these results show that a range of HPgV-1 genotypes are circulating in Tanzania and Equatorial Guinea, clustering to published genotypes 1, 2 and 5.

### Effect of HPgV-1 positivity on systemic cytokine and chemokine levels

**Fig. 6:**
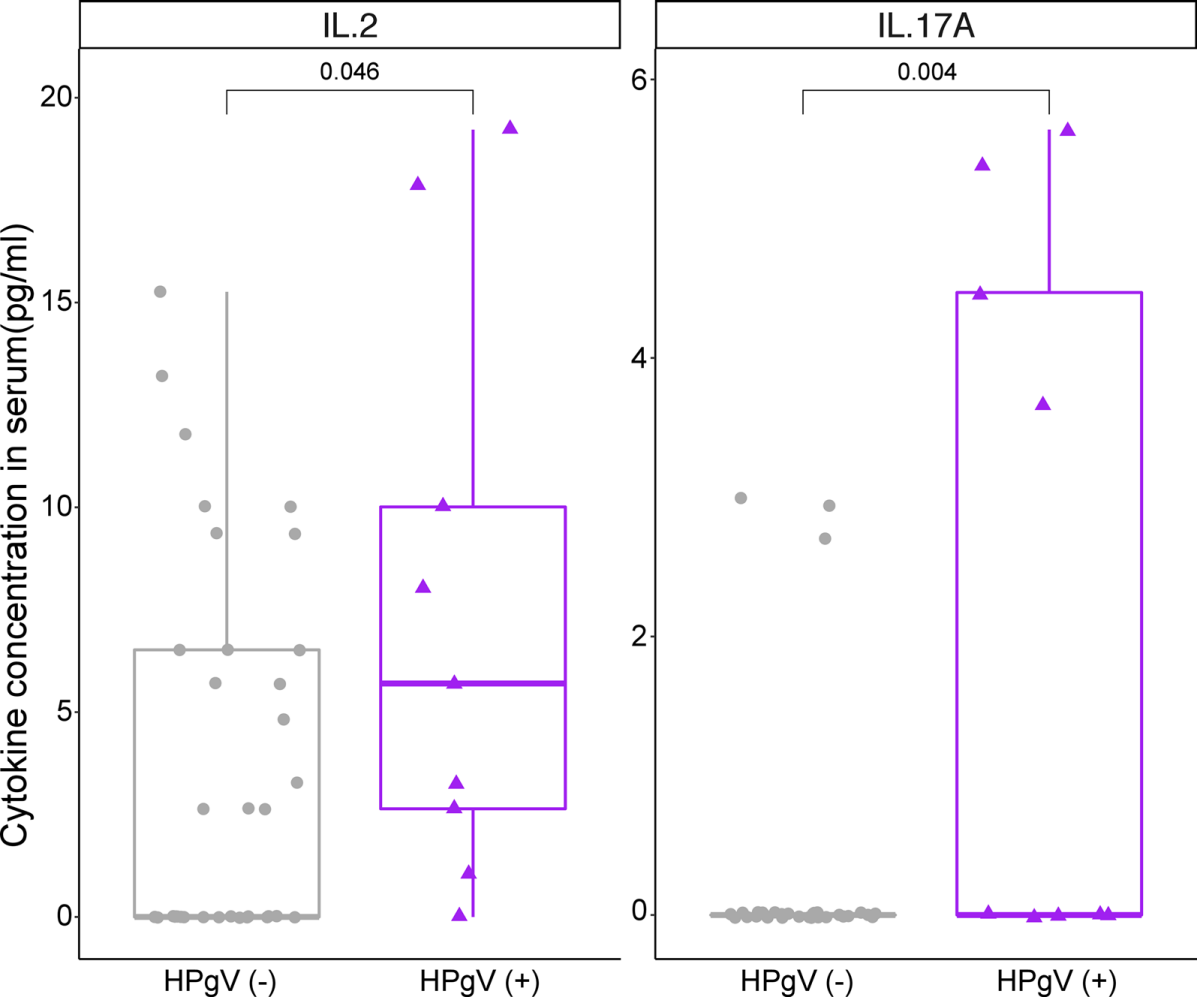
HPgV-1 infection is associated with increased systemic levels of IL-2 and IL-17A. Cytokine, chemokine and growth factors levels were analysed by Luminex and levels compared between HPgV-1 negative (5′ UTR-**,** grey, n = 35**)** and HPgV-1 positive (5′ UTR + , purple, n = 9) volunteers. Absolute serum concentrations levels (pg/ml) of Interluekin-2 (IL-2) and Interluekin-17A (IL-17A) at samples taken before vaccination are shown. Significantly higher IL-2 and IL-17A are seen in the HPgV-1^**+**^ compared to the HPgV-1^**−**^. Wilcoxon rank sum test was used to determine significance (*P* value * < 0.05) which are indicated on top of top for each group comparison

To dissect whether ongoing HPgV-1 infection affects cytokine and chemokine levels in serum, we measured 45 cytokines, chemokines and growth factors in a subset of 44 volunteers from BSPZV3a (HIV-1 negative only) and EGSPZV2. 23 cytokines, chemokines and growth factors were detected above their pre-defined lower limits of detection (Additional file 5: Table 1). Although there was a trend of overall higher cytokine levels in HPgV-1 infected individuals (Additional file 2:  Fig. 2), only IL-2 and IL-17A reached significance levels (Fig. 6). There was no statistically significant difference in cytokine and chemokine levels when HPgV-1 high and low viremic individuals were compared (data not shown). Also, we could not find differences in chemokine and cytokine levels when comparing the different HPgV-1 genotypes (data not shown). Taken together, these data suggest that the presence of HPgV-1 infection increases IL-2 and IL-17A levels in peripheral blood.

### Effect of HPgV-1 infection status on PfSPZ vaccine-induced humoral immune response

**Fig. 7:**
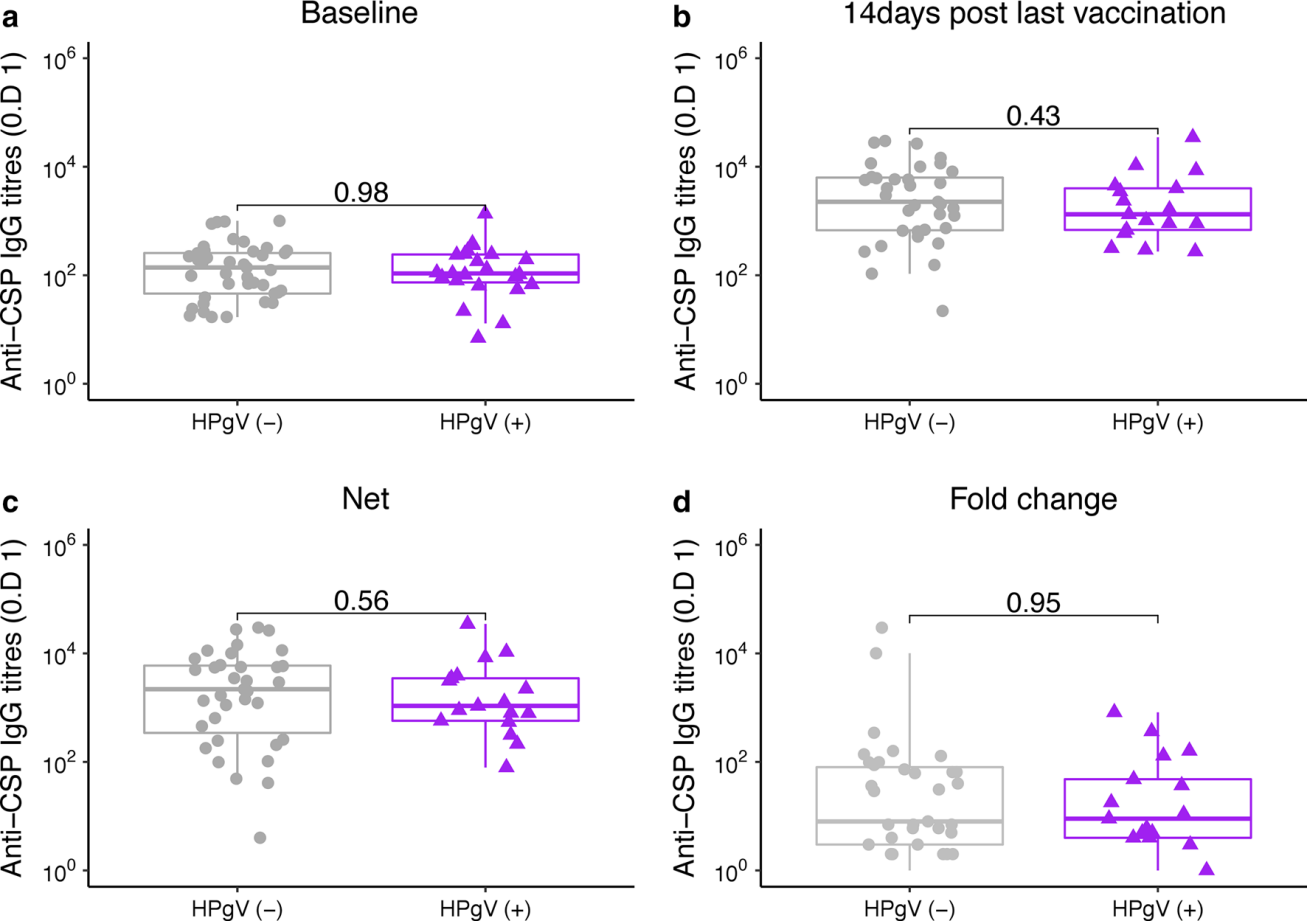
HPgV-1 infection status does not impact on anti-PfCSP antibody titres. Total IgG antibodies recognizing full length PfCSP were measured by ELISA in HPgV-1 negative (5′ UTR-**,** grey), HPgV-1 positive (5′ UTR + , purple) volunteers. **a** Shows baseline (pre-vaccination) anti-PfCSP IgG levels of HPgV-1 negative (n = 47) compared to HPgV-1 positive (n = 23) volunteers. **b** Anti-PfCSP IgG levels at 14 days past last vaccination in HPgV-1 positive individuals (n = 17) versus the HPgV-1 negative (n = 37) group. **c**, **d** Comparison of vaccine-induced changes in anti-PfCSP IgG titres as net responses (14 days post last immunization—baseline) as well as fold (14 days post last vaccination/baseline)**.** Only vaccinated individuals were included for 14 days post last immunization, net and fold change responses**.** One HPgV-1^+^ individual was not included in these subsequent analyses due to missing antibody data. Log anti-PfCSP titres expressed in arbitrary units are shown**.** Each point represent an individual, box plot with horizontal bar show median values for each group. Statistical significance was calculated by using Wilcoxon rank sum test (*P *value * < 0.05). *P* values are indicated on top for each group comparison

IL-2 and IL-17A might contribute to differentiation of naïve B cells into plasma cells and support the survival of activated B cells [46, 47]. We examined the potential of HPgV-1 infection to impact on PfSPZ vaccine-induced humoral immunity. PfCSP is the most immuno-dominant protein recognized after PfSPZ vaccination. Anti-PfCSP titres were measured at baseline in all volunteers (n = 70) and 14 days past last vaccination in all vaccinated volunteers (n = 54) participating in BSPZV2, BSPZV3a and EGSPZV2 (Fig. 7 A-B). Similar results were observed when PfSPZ vaccine-induced antibody responses were analysed as net increase (titres at 14 days post last vaccination minus baseline titres) (Fig. 7 C) and as fold change (titres 14 days past last vaccination divided by baseline titres) (Fig. 7 D). There was no significant correlation between HPgV-1 infection status and anti-PfCSP antibody titre at baseline and after vaccination.

### Effect of HPgV-1 infection on PfSPZ vaccine efficacy

**Fig. 8:**
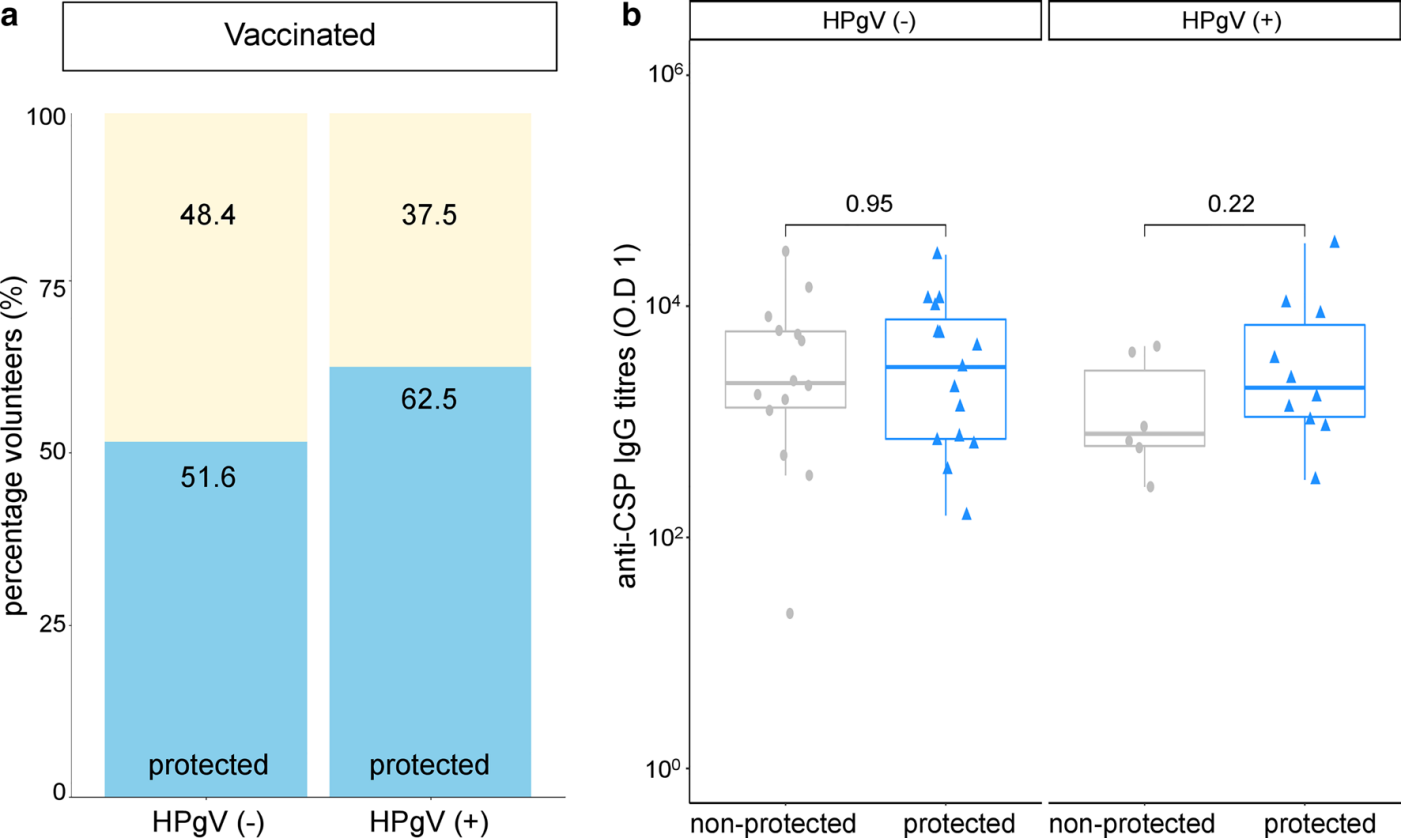
HPgV-1 infection does not influence *P. falciparum* pre-patent periods and parasite multiplication rates during CHMI. Parasitemia was determined in whole blood by qPCR and thick blood smear microscopy (TBS). The analysis included only placebo participants, positive and negative for HPgV-1. **a** Shows log-fold change of parasitemia in 48 h between HPgV-1 negative (5′ UTR-, grey, n = 13) and HPgV-1 positive (5′ UTR + , purple, n = 7) volunteers. **b** Comparison of days post CHMI to malaria positivity by microscopy in HPgV-1 negative (5′ UTR-**,** grey, n = 11) and HPgV-1 positive (5′ UTR + , purple, n = 7). **c** HPgV-1 viral loads before (red) and 28 days post CHMI (green) in HPgV-1 infected individuals. Each point represents an individual, box plots show data distribution with horizontal bar denoting viral load at each visit. Lines connect viremia levels in individuals found positive for HPgV-1 on both time points. Geometric means were compared between groups and unpaired t-test was used to calculate significance. Horizontal bars represent mean with standard deviation Wilcoxon rank sum test was used to compare viremia levels before and after CHMI. P-values are indicated on top of each comparison

The high prevalence of HPgV-1 positive volunteers in our cohort allowed us to investigate a potential impact of ongoing viral infection during PfSPZ vaccination on vaccine-induced protection. Protective efficacy of the vaccine was evaluated by presence or absence of asexual blood-stage parasitemia following homologous PfSPZ challenge (CHMI) (Additional file 3: Fig. 3). While none of the placebo-receiving participants was protected (0/20), the overall protection in the vaccinated group was 55% (26/47). The HPgV-1 prevalence was comparable in these two groups (placebos and vaccines) with 35% (7/20) versus 34% (16/47), respectively, suggesting that HPgV-1 infection does not facilitate protection against CHMI. We further compared the CHMI protection levels in HPgV-1 positive and negative participants in the vaccinated group only (Fig. 8a). HPgV-1 positive vaccinees showed slightly higher protection levels after CHMI (62.5%; 10/16) compared to HPgV-1 negative individuals (51.6%; 16/31). We also assessed anti-PfCSP antibodies titres at the peak response which is 14 days past last vaccination. Slightly higher anti-PfCSP levels were seen in protected compared to non-protected individuals (Fig. 8b), without reaching statistical significance. PfCSP antibody levels tended to be lower in the HPgV-1 positive individuals (Fig. 8b).

### Interaction of HPgV-1 and controlled human malaria infection induced asexual blood-stage parasites

**Fig. 9:**
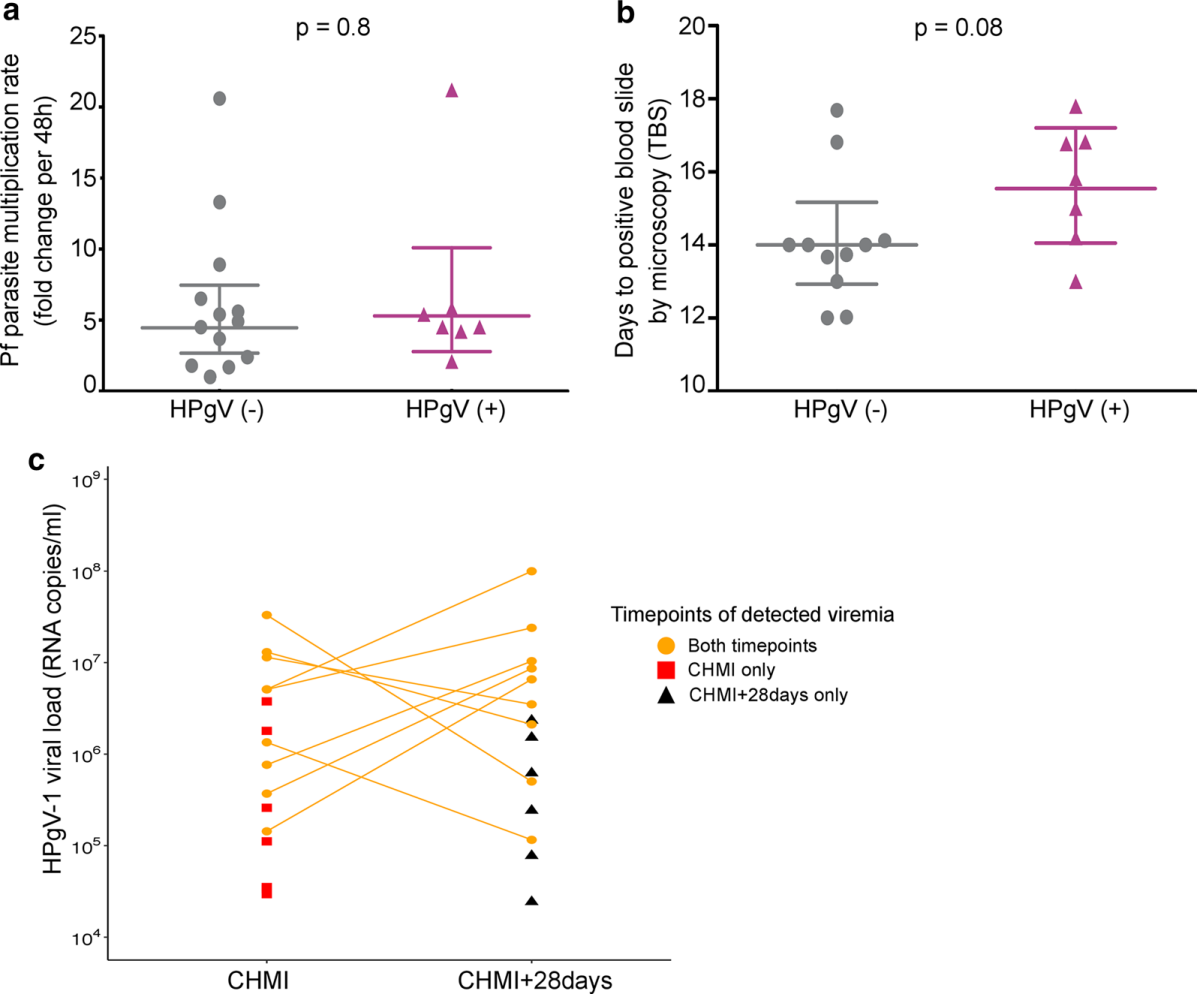
Association of HPgV-1 infection status with PfSPZ CHMI outcome and anti-CSP titers in immunized volunteers. Individuals were treated with either normal saline (placebo) or PfSPZ Vaccine (vaccinees). Presence or absence of malaria parasites was determined in whole blood by thick blood smear microscopy (TBS) and confirmed by qPCR. Total IgG antibodies recognizing full length PfCSP were measured by ELISA. **a** Proportion of non-protected (cream) and protected (blue) in vaccinated volunteers with and without HPgV-1 infection. Proportions are indicated inside the bar and volunteer numbers on top. **c** Total anti-CSP IgG levels at 14 days past last vaccination in the protected (malaria negative) and non-protected (malaria positive) groups, with and without HPgV-1 infection. Log anti-PfCSP titres expressed in O.D units are shown**.** Each point represent an individual, and box plot with horizontal bar show median values for each group. Chi square with Yates correction was used for group comparisons of categorical values (*P < 0.05). Wilcoxon rank sum test was used to compare anti-CSP titres in the two groups. *P* values are indicated on top of each comparison

HPgV-1 co-infection has been associated with favourable clinical outcomes in HIV-1 and Ebola co-infected individuals [21–23]. So far, the HPgV-1 impact on *P. falciparum* infection and immunity is unknown. We evaluated parasite multiplication rates and pre-patent periods in the placebo volunteers only, that have not been vaccinated (n = 20) undergoing CHMI. Comparable asexual blood-stage multiplication rates and pre-patent periods were observed between HPgV-1 positive and negative individuals (Fig. 9a, b).

### Effect of CHMI on HPgV-1 viremia levels

*P. falciparum* infection is known to impact viremia levels of some common viruses like HIV-1 and EBV [48, 49]. We therefore evaluated the effect of an acute *P. falciparum* infection on HPgV-1 viremia by comparing the viral load before and 28 days post CHMI in 21 eligible trail participants. Detectable levels of HPgV-1 at both time points were quantified in 9 individuals; 5 of those showed an increased HPgV-1 viral load and the other 4 had decreased viremia post CHMI (Fig. 9c). In addition, 12 trial participants were HPgV-1 positive only for one of the two tested time points; 6 were positive before CHMI and 6 were positive at 28 days post CHMI (Fig. 9c) (Additional file 4:  Fig. 4 showing four representative volunteers individually).

## Discussion

The role of chronic asymptomatic viral infections in modulating immune responses in health and disease is increasingly appreciated [50]. The present study sought to better understand the prevalence and genotype distribution of HPgV-1 in East and West-central Africa. We aimed to investigate the potential influence of HPgV-1 infection on experimental malaria vaccine-induced humoral immunity and vaccine-induced protection. By studying a cohort of volunteers undergoing CHMI, we were in a unique position to investigate if an acute malaria episode has an impact on HPgV-1 viremia in chronically infected volunteers.

In a first step, we used an unbiased approach by generating RNA-seq data to identify prevalent viruses circulating in peripheral blood of our BSPZV1 study volunteers that lead to the identification of HPgV-1 as highly present in this group. Confirmation by RT-PCR was possible only in 2/8 HPgV-1 positive volunteers. Several factors might have contributed to these discrepancies in results, such as the volume and type of samples used. 300 ul of plasma was used as starting material for RNA extraction and RT-PCR, while roughly 10 times more whole blood was applied as starting material for the RNA-seq data generation. Also, the variation in HPgV-1 viremia levels between serum and cellular components have been demonstrated previously [19, 25]. The drawbacks of RT-PCR based methods over deep RNA sequencing methods in virus identification have been previously reported. To overcome these limitations, the combination of qPCR working with predefined primers and unbiased deep sequencing approaches is recommended [51, 52]. The unbiased virome analyses were important for focusing our analyses on HPgV-1 in larger cohorts in East and West Africa.

The overall prevalence of HPgV-1 in our main study cohort was 29.2%, roughly the same for Tanzania and Equatorial Guinea. The prevalence reported here is likely underestimated as we observed fluctuations of HPgV-1 viral loads longitudinally, with some volunteers showing HPgV-1 positivity in one, two or all three time points assessed**.** These detection variations might indicate either viral clearance or continuously ongoing viral replication with viremia fluctuations sometimes below the lower detection limit of our RT-qPCR assay [19, 25]. We did not detect HPgV-2 RNA in any of our volunteers but we cannot completely exclude the possibility of the presence of circulating HPgV-2 as antibody titers against the HPgV-1 and HPgV-2 E2 envelope proteins were not measured. Our study focussed on the potential impact of HPgV-1 on PfSPZ vaccine induced humoral responses and protection, thus HIV-1 positive individuals of the BSPZV3a study were excluded from the HPgV-1 association analyses. It is well known that HIV-1 infection negatively impacts immunity in widely used routine vaccines [53].

Similar to a study in Mexico, we observed two broad groups, low and high, of HPgV-1 viremic individuals, defined by a cut off value of 600,000 RNA copies/ml. This observation likely reflects the different viral replication states within infected volunteers [22]. We observed similar numbers of high and low viremic individuals, who are infected with HPgV-1 genotype 2. The potential role of a distinct viral genotype on this pattern remains unclear, given the small number of volunteers in this study and limited heterogeneity of the detected HPgV-1 genotypes.

Currently, 7 HPgV-1 genotypes are described globally [54, 55] and some of these genotypes have been implicated in varied clinical outcomes [23, 26, 56]. HIV-1/HPgV-1 co-infection studies revealed lower CD4 T cell counts in individuals infected with HPgV-1 genotype 2a than genotype 2b [56, 57] and higher HPgV-1 viral loads in individuals with genotype 1 compared to genotypes 2a and 2b [58]. Higher serum levels of IFN-ɣ were described in HIV-1 positive women co-infected with genotype 2 compared to genotype 1 [23].

Phylogenetic analyses in our cohort demonstrated the presence of genotype 1 (n = 2, 7.1%) and 2 (n = 24, 92.3%). Most of our genotype 2 strains clustered within group 2a, originally described from Venezuela. Genotype 1 and 2 have been previously reported in Tanzania but there are no published data available for Equatorial Guinea [59, 60]. The predominance of genotype 2 in our study is somewhat surprising. Given the diverse geographic origin of our volunteers recruited from East and West-central Africa, we had expected to find higher HPgV-1 genetic diversity. Studies in neighbouring countries including Cameroon, the Democratic Republic of Congo and Gabon revealed a high prevalence of genotype 1 [61–65]. Genotypes 2 and 5 were also seen when phylogenetic studies included molecular markers other than 5′ UTR region like envelope protein 1 (E1), non-structural protein 3 (NS3) and non-structural protein 5A [62, 66]. The limitations of amplification of the 5′ UTR, a highly conserved region, to discriminate closely related isolates is known [67]. Due to its high variability, E2 provides better genotyping resolution compared to 5′ UTR. We amplified and sequenced the E2 region from subjects with high viremia levels in serum (n = 9). Based on the E2 sequences, these 9 isolates clustered with strains described elsewhere in Africa. It is possible that the failure to amplify E2 from all volunteers positive by 5′ UTR detection is either due to the low sensitivity of the assay used or the high genetic diversity of the E2 region [42]. While it is known that the detection of HPgV-1 based on amplification of the E2 region is highly specific, it requires higher amounts of RNA input [42] and individuals with low HPgV-1 viremia are likely missed. Alternatively, it is possible that E2 genetic variants could not be amplified with the primers used due to nucleotide sequence mismatch. The E2 region is highly variable and this diversity contributes to structural, functional and immunogenic properties of the virus [68]. The inconsistent genotyping results of isolates 6EG and 14EG based on 5′ UTR and E2 amplification might be resolved by whole genome sequencing of the virus. Vitrenko et al., reported similar findings in samples from Ukrainian females donating fetal tissues [67].

Cytokines, chemokines and growth factors are important for inter-cellular communication and regulation of immune processes [69]. Any changes in levels of these immune mediators can act as markers of inflammation, immunity or vaccine uptake [26, 70, 71]. We therefore investigated if altered levels of cytokines and chemokines unique to ongoing HPgV-1 infection could be identified. We analysed serum samples taken at baseline for 45 cytokines in a Luminex platform. Volunteers with chemokine and cytokine levels above the lower limit of detection were stratified according to the HPgV-1 infection status. Of all 23 differentially detected cytokines and chemokines, IL-2 and IL-17A were significantly higher in HPgV-1 positive compared to HPgV-1 negative individuals.

IL- 2 is an essential survival factor for T and B lymphocytes [47, 72] and induces the development and survival of regulatory CD4 T cells critical for the maintenance of immune tolerance [73]. Fama et al., showed increased levels of circulating soluble IL-2 receptor (sIL-2R) in HPgV-1 positive volunteers but the authors did not quantify IL-2 levels [74]. The increased concentrations of IL-2 seen amongst the HPgV-1 positive individuals could be linked to either on-going antiviral immunity [75] or serves as a survival mechanism used by the virus to establish persistence in immune cells. A similar mechanism has been described in the apicomplexan pathogen *Theileria parva* that infects T and B lymphocytes in cattle [76]. Contrary to our observations are results from HPgV-1/HIV-1 coinfection studies which have shown reduced T-cell activation and IL-2 release in coinfected individuals [77, 78]. The HPgV-1 envelope protein 2 (HPgV1-E2) has been implicated in these outcomes, due to its ability to inhibit T cell-receptor mediated signalling and IL-2 signalling pathways [77, 78].

IL-17A induction has been associated with bacterial, fungal, autoimmune and inflammatory diseases [79]. IL-17A stimulates production of chemokines such as monocyte chemoattractant protein-1 which mediates tissue infiltration of monocytes. The role of IL-17A in the context of HPgV-1 infection is unknown. However, in other viral infections like HIV-1 and Hepatitis C, IL-17A has been shown to promote T-cell mediated anti-viral responses through activation and recruitment of dendritic cells, monocytes and neutrophils [80, 81]. Other cytokines and chemokines which could be detected, albeit not significantly different in HPgV-1 positive individuals included SCF (lower) and IL-1beta, IL-12p70, MCP-1, LIF, VEGF.A, HGF and TNF-α (higher). BDNF, EGF, Eotaxin, GRO-alpha, IFN-ɣ, IL-7, IP-10, MIP1-a, Mip-1b, PDGF.BB, PIGF.1, RANTES, SDF-1a, and VEGF.D were comparable between the two groups. The levels of these measured cytokines and chemokines are within ranges previously reported [26, 27]. While most of the previous HPgV-1 studies had focused on at risk populations, particularly on HIV-1 positive persons, our investigations are in healthy individuals [26, 74], therefor some of the observed differences could be due to health status.

Here, we observed lower, albeit not statistically significant, median anti-PfCSP titres in the HPgV-1 positive versus the negative group at baseline and 14 days past last vaccination. These observations mirror findings by Avelino-Silva et al., who showed no association between HPgV-1 infection status/viremia with yellow fever specific neutralizing antibody titres in HIV-1 positive individuals immunized with yellow fever vaccine [82]. While studies have extensively tried to understand potential inhibition mechanisms induced by HPgV-1 (and other Flaviviruses) on T cell activation [77, 83], activation pathways that might be affected in B cells are less explored. It is also possible that the effect of HPgV-1 viruses on immune responses against vaccines is negligible when studied singly, but this impact is significantly synergized in the presence of other, co-infecting viruses like EBV, CMV and HSV [85, 86]. Hence, the potential role played by the combined human virome in shaping vaccine-induced responses in different populations needs to be further explored in larger cohorts.

Clinically silent, chronic viral infections are known to modulate host immunity [16] and in turn, acute co-infections are known to drive the re-activation of asymptomatic viral infections [49]. Several viruses, like HIV-1, Ebola and HCV have been implicated in the pathogenesis and clinical outcome of ongoing malaria infections through a range of different mechanisms [84–86]. It has been suggested that HIV-1 infections worsen *P. falciparum* presentations by depleting the CD4 T-cell compartment, essential for driving malaria-specific antibody responses and for clearance of malaria infected red blood cells [84]. In contrast, better survival outcomes have been reported in Ebola infected individuals with *P. falciparum* co-infections [85]. Reports have also suggested delayed emergence of *P. falciparum* asexual blood-stages in Gabonese individuals chronically infected with HCV [86]. Thus, we studied the impact of HPgV-1 positivity on asexual *P. falciparum* parasitemia and multiplication rates during CHMI. Vice versa, we also looked for the first time at the impact of PfSPZ vaccination and PfSPZ challenge on HPgV-1 viremia. We could not find evidence of an association between HPgV-1 infection status and asexual blood-stage parasite multiplication rates after CHMI. A slight trend towards longer pre-patent period was seen in HPgV-1 positive individuals. HPgV-1 positivity appears to increase malaria vaccine-induced protection, since slightly higher proportion of CHMI protected individuals were HPgV-1 positive (62.5% vs 51.6%). However, our study is limited by the sample size and further investigations with larger cohorts are required to corroborate these findings. Importantly, PfSPZ vaccination and PfSPZ challenge did not impact HPgV-1 viremia levels in our cohort suggesting that the conduct of CHMI is safe in HPgV-1 infected volunteers.

## Conclusions

Notable effects have been reported in HPgV-1 co-infections with other RNA viruses such as HIV-1 and Ebola. Although our study is constrained with limited sample size, we have highlighted the epidemiology and genetic distribution of HPgV-1 in areas endemic for malaria. We have reported for the first time HPgV-1 genotype distribution in Equatorial Guinea. We examined the potential influence of HPgV-1 infection status on PfSPZ vaccine-induced PfCSP-antibody titres and CHMI outcome without finding any striking correlation. Our study provides first time evidence that intravenous vaccination using large numbers of attenuated *P. falciparum* sporozoites and CHMI does not increase HPgV-1 viremia in already infected volunteers.

## Supplementary information

**Additional file 1.** **Fig. 1** Flow chart of volunteers included in virome pilot study and analyses pipeline. A) Flow chart of volunteers included in virome pilot study and analyses. Samples for transcriptomic studies were selected from a subset of volunteers of BSPZV-1 (n=28). RNA sequencing was performed and, differential gene expression and blood transcriptome modules were analysed. Non –human reads data was used for virome analyses. B) Virus identification: Pilot virome study analysis pipeline-“Bagamoyo viromescan” i) Non-human (un-mapped reads) were searched for “suspected” viral hits in NCBI database containing more than 7424 viral genomes using bowtie 2. ii) Removal of low quality and complexity reads as well as reads mapping to human genome, transcriptome and repeat regions by bowtie 2, knead data and tandem repeat finder algorithms respectively. iii) Search for viral hits in the “clean” viral reads using virome scan and Taxonomer and for viral proteins using Diamond tool. iv) The non-human unmapped reads were also analysed by Fast virome explorer, without filtering host reads to allow the identification of endogenous retroviral elements and other viruses that may have been missed by Taxonomer and viromescan. C) Viral confirmation: i) Pre-selection criteria for suspected viral hits by each tool ii) In-silico confirmation of suspected viral hits through blasting in NCBI and mapping against specific viral whole genomes in geneous tool; and removal of viral contaminants. iii) Laboratory confirmation of viruses by reverse transcription polymerase chain reaction.

**Additional file 2.** **Fig. 2** Impact of HPgV-1 infection on systemic cytokines and chemokines. Absolute cytokines, chemokines and growth factor levels at baseline are shown based on HPgV status: HPgV-1 negative (-), grey (n=35) and HPgV-1 positive (+), purple (n=9). Comparable median levels of Brain derived neutrophil factor (BDNF), Epidermal growth factor (EGF), Eosinophil chemoattractant cytokine (Eotaxin/ CCL11), Growth regulated oncogene-alpha (GRO-alpha), Interferon gamma (IFN-ɣ), Interluekin-7 (IL-7), Interferon gamma induced protein- 10 (IP-10), Macrophage inflammatory protein 1-alpha(MIP1-a), MIP1-b (Macrophage Inflammatory protein 1-beta), Platelet derived growth factor BB (PDGF.BB), Placental growth factor (PIGF.1), Regulated on activation normal T cells and excreted (RANTES), Stromal derived factor 1 alpha (SDF-1a) , and Vascular endothelial growth factor D (VEGF.D); Lower median levels of Stem cell factor (SCF); and higher median levels of Monocyte chemoattractant protein 1 (MCP-1), Leukemia inhibitory factor (LIF), Vascular endothelial growth factor A (VEGF.A), Hepatocyte growth factor (HGF) and Tumor Necrosis Factor-alpha (TNF-α) in the HPgV-1 positive individuals. Cytokines, chemokines and growth factors with values above their predefined lower detection limit were considered substantial. Wilcoxon rank sum test was used to compare the two groups and P-values are indicated on top for each comparison

**Additional file 3. Fig. 3** Vaccine trial design and procedures. Volunteers are enrolled and randomized into placebo (black icons) and vaccine groups (green icons). Immunized with specified dose of radiated-attenuated whole sporozoites or whole sporozoites with antimalarial drug (V1, V2; V3 etc.) and subsequently challenged with homologous PfSPZ parasites used for vaccination (CHMI). Volunteers are monitored in a controlled setting up to 21 days with venous blood drawn daily to monitor presence (malaria positive, not protected) or absence (malaria negative, protected) of asexual blood-stage parasitemia. All volunteers were treated with an anti-malarial drug either once turning TBS positive or at day 28 after start of CHMI. Further monitoring of volunteers occurred at 56 days post CHMI. HPgV-1 infection was evaluated in plasma samples from the time points highlighted in blue.

**Additional file 4. Fig. 4** HPgV-1 RNA positivity and viremia across study visits (Baseline, CHMI and CHMI+28) in Tanzania and Equatorial Guinea. HPgV-1 viral plasma RNA was measured by RT-qPCR at baseline (pre-vaccination), before (CHMI) and 28 days post immunization (CHMI+28 days ) in Tanzanian (n=45) and Equatorial Guinean (n=51) volunteers. Here four volunteers from the whole cohort are displayed as a representation. The figure depicts inter-individual variability in HPgV-1 RNA detection with some individuals negative or positive at one, two or all three measured time points. Log 10 viral loads are plotted on the y-axis and the time points on the x-axis. Each square plot represents an individual with volunteer identification numbers indicated on top. Each dot corresponds to a single time point connected to the next by a solid line. The horizontal dashed line indicates the threshold value of zero viremia.

**Additional file 5.** **Table 1** Sensitivity and standard curve ranges for the 45 cytokines, chemokines and growth factors analysed in this study. The tables shows the 45 cytokines, chemokines and growth factors their sensitivities and standard curve ranges as provided by manufacturer.

## Data Availability

Data are available from the corresponding author upon reasonable request.

## Abbreviations

CHMI: Controlled human malaria infection
CSP: Circumsporozoite protein
E1/2: Envelope glycoproteins (1 and 2)
HPgV: Human pegivirus
IVT: In vitro transcription
LEfSe: Linear discriminant analysis effect size
NK: Natural killer cells
NS5A: Non-structural protein 5A
PfCSP: *Plasmodium falciparum* Circumsporozoite protein
PfSPZ: Plasmodium falciparum sporozite
PMR: Parasite Multiplication Rate
RNaseP: Ribonuclease P
SSA: Sub-Saharan Africa
TBS: Thick blood smear
UTRs: Untranslated regions
