# Supplementary figures and images for: Role of human Pegivirus infections in whole Plasmodium falciparum sporozoite vaccination and controlled human malaria infection in African volunteers
Source: Virol J. 2021 Jan 26;18:28. doi: 10.1186/s12985-021-01500-8 (PMC7837505; doi:10.1186/s12985-021-01500-8)

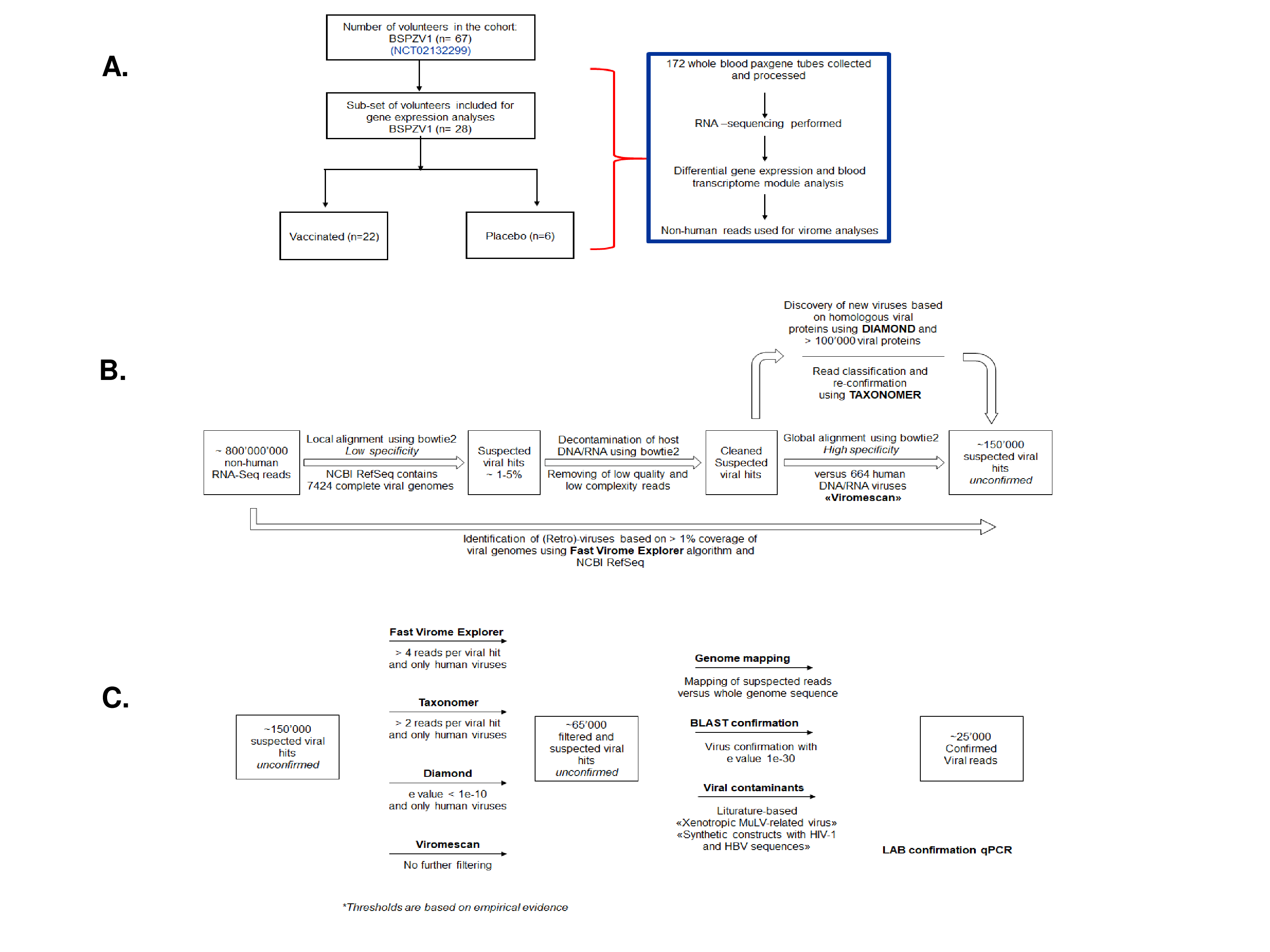

Supplement: Supplementary file 1 — Additional file 1. Fig. 1 Flow chart of volunteers included in virome pilot study and analyses pipeline. A) Flow chart of volunteers included in virome pilot study and analyses. Samples for transcriptomic studies were selected from a subset of volunteers of BSPZV-1 (n=28). RNA sequencing was performed and, differential gene expression and blood transcriptome modules were analysed. Non –human reads data was used for virome analyses. B) Virus identification: Pilot virome study analysis pipeline-“Bagamoyo viromescan” i) Non-human (un-mapped reads) were searched for “suspected” viral hits in NCBI database containing more than 7424 viral genomes using bowtie 2. ii) Removal of low quality and complexity reads as well as reads mapping to human genome, transcriptome and repeat regions by bowtie 2, knead data and tandem repeat finder algorithms respectively. iii) Search for viral hits in the “clean” viral reads using virome scan and Taxonomer and for viral proteins using Diamond tool. iv) The non-human unmapped reads were also analysed by Fast virome explorer, without filtering host reads to allow the identification of endogenous retroviral elements and other viruses that may have been missed by Taxonomer and viromescan. C) Viral confirmation: i) Pre-selection criteria for suspected viral hits by each tool ii) In-silico confirmation of suspected viral hits through blasting in NCBI and mapping against specific viral whole genomes in geneous tool; and removal of viral contaminants. iii) Laboratory confirmation of viruses by reverse transcription polymerase chain reaction. [file 12985_2021_1500_MOESM1_ESM.png]

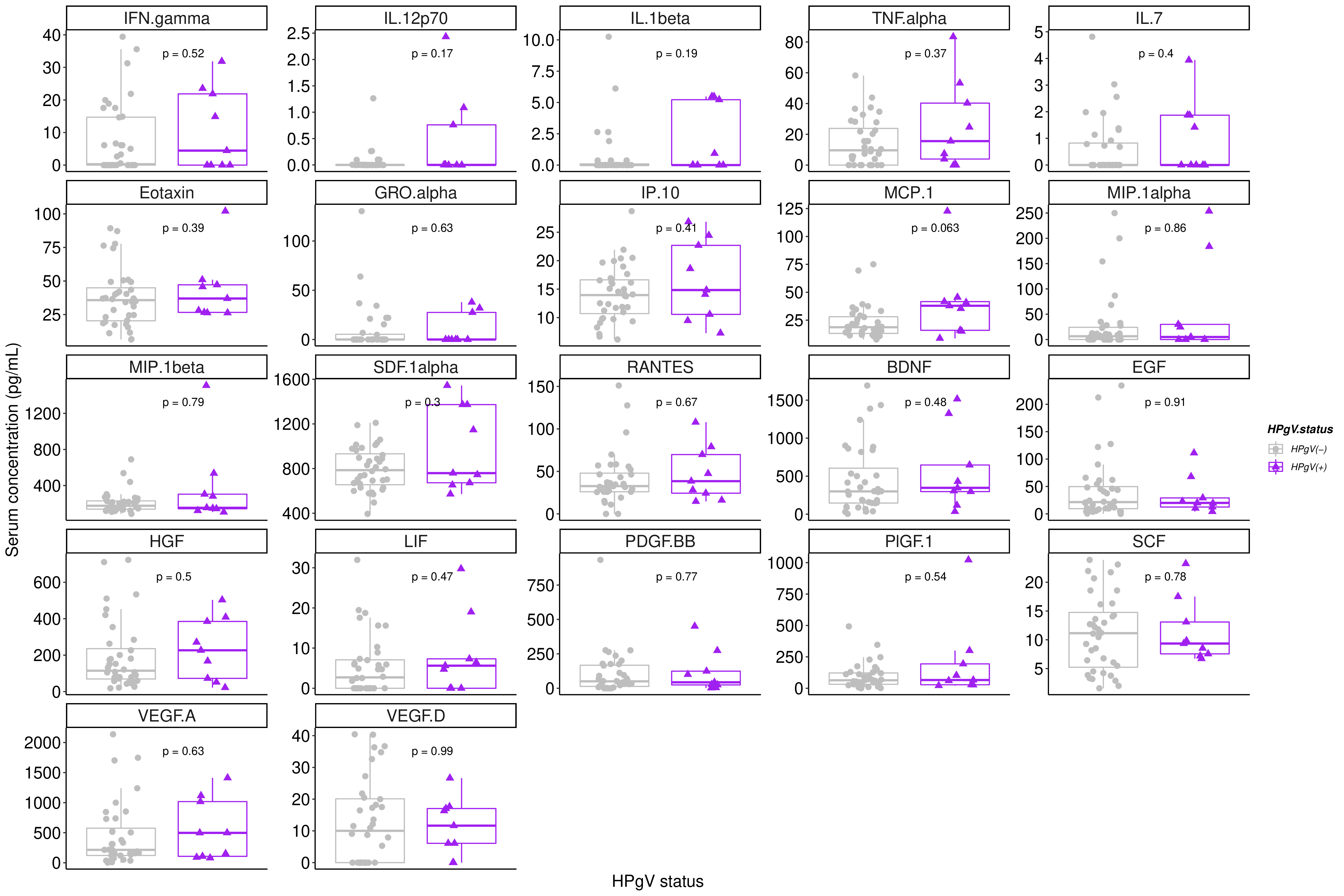

Supplement: Supplementary file 2 — Additional file 2. Fig. 2 Impact of HPgV-1 infection on systemic cytokines and chemokines. Absolute cytokines, chemokines and growth factor levels at baseline are shown based on HPgV status: HPgV-1 negative (-), grey (n=35) and HPgV-1 positive (+), purple (n=9). Comparable median levels of Brain derived neutrophil factor (BDNF), Epidermal growth factor (EGF), Eosinophil chemoattractant cytokine (Eotaxin/ CCL11), Growth regulated oncogene-alpha (GRO-alpha), Interferon gamma (IFN-ɣ), Interluekin-7 (IL-7), Interferon gamma induced protein- 10 (IP-10), Macrophage inflammatory protein 1-alpha(MIP1-a), MIP1-b (Macrophage Inflammatory protein 1-beta), Platelet derived growth factor BB (PDGF.BB), Placental growth factor (PIGF.1), Regulated on activation normal T cells and excreted (RANTES), Stromal derived factor 1 alpha (SDF-1a) , and Vascular endothelial growth factor D (VEGF.D); Lower median levels of Stem cell factor (SCF); and higher median levels of Monocyte chemoattractant protein 1 (MCP-1), Leukemia inhibitory factor (LIF), Vascular endothelial growth factor A (VEGF.A), Hepatocyte growth factor (HGF) and Tumor Necrosis Factor-alpha (TNF-α) in the HPgV-1 positive individuals. Cytokines, chemokines and growth factors with values above their predefined lower detection limit were considered substantial. Wilcoxon rank sum test was used to compare the two groups and P-values are indicated on top for each comparison [file 12985_2021_1500_MOESM2_ESM.png]

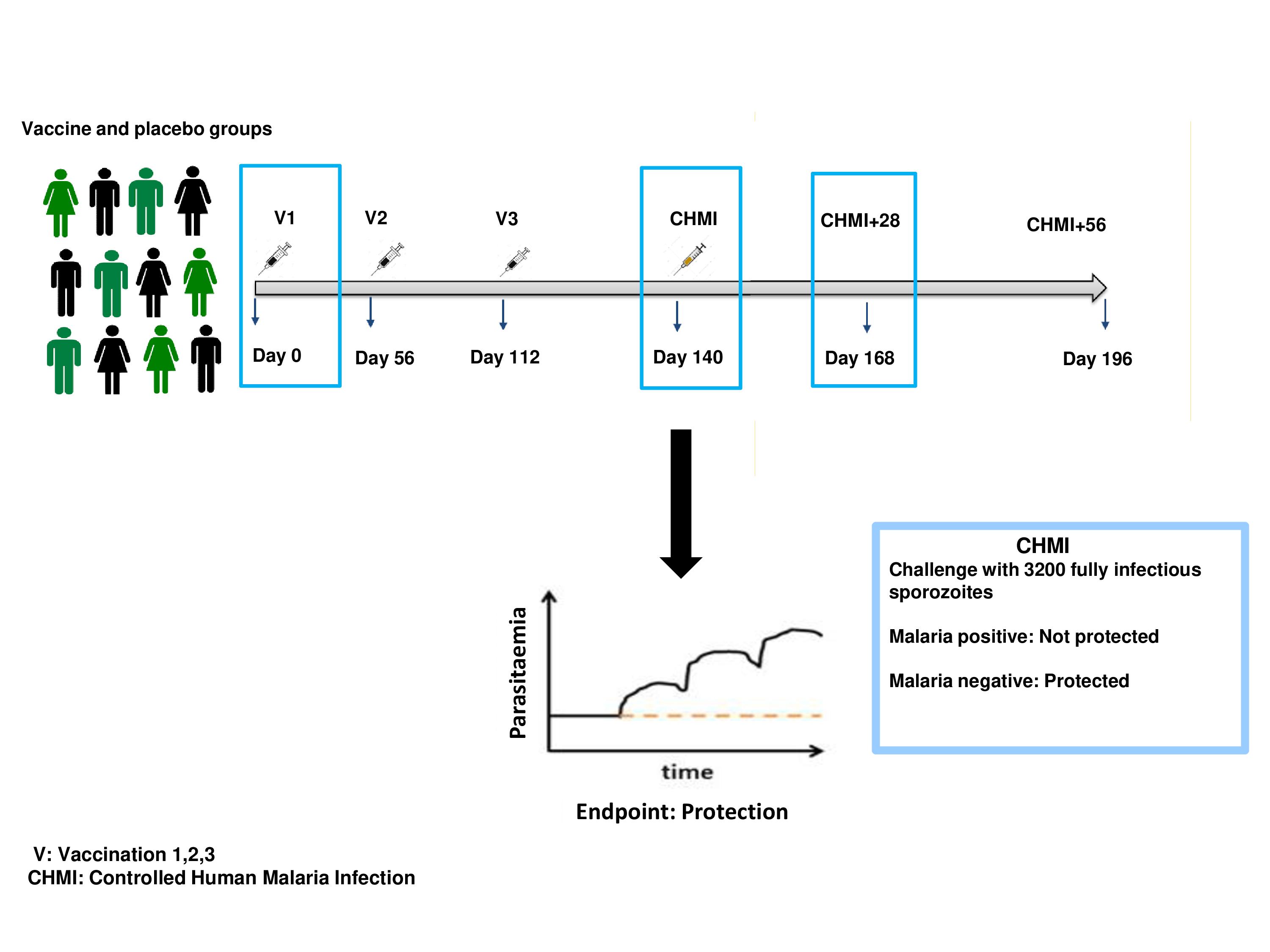

Supplement: Supplementary file 3 — Additional file 3. Fig. 3 Vaccine trial design and procedures. Volunteers are enrolled and randomized into placebo (black icons) and vaccine groups (green icons). Immunized with specified dose of radiated-attenuated whole sporozoites or whole sporozoites with antimalarial drug (V1, V2; V3 etc.) and subsequently challenged with homologous PfSPZ parasites used for vaccination (CHMI). Volunteers are monitored in a controlled setting up to 21 days with venous blood drawn daily to monitor presence (malaria positive, not protected) or absence (malaria negative, protected) of asexual blood-stage parasitemia. All volunteers were treated with an anti-malarial drug either once turning TBS positive or at day 28 after start of CHMI. Further monitoring of volunteers occurred at 56 days post CHMI. HPgV-1 infection was evaluated in plasma samples from the time points highlighted in blue. [file 12985_2021_1500_MOESM3_ESM.png]

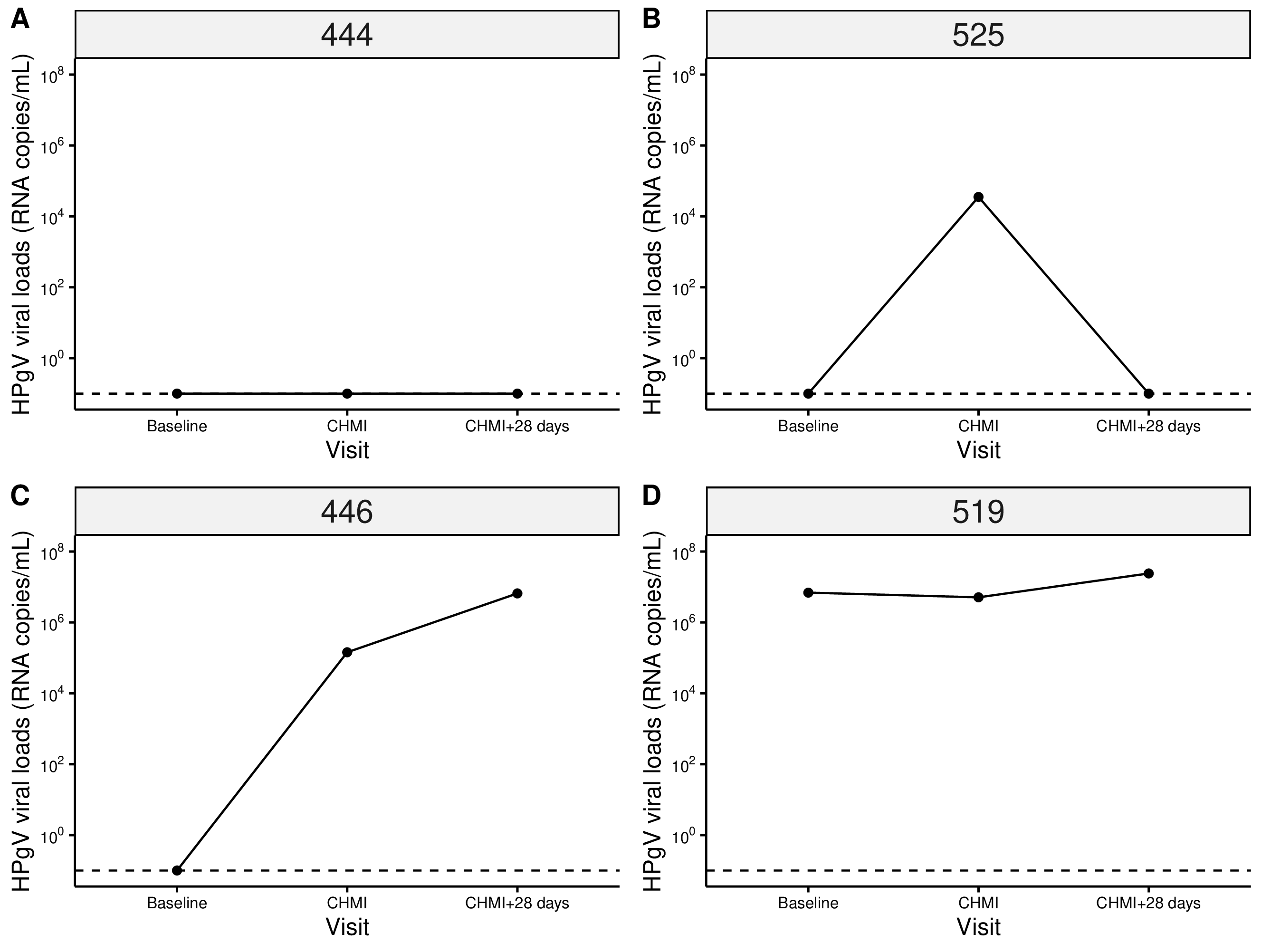

Supplement: Supplementary file 4 — Additional file 4. Fig. 4 HPgV-1 RNA positivity and viremia across study visits (Baseline, CHMI and CHMI+28) in Tanzania and Equatorial Guinea. HPgV-1 viral plasma RNA was measured by RT-qPCR at baseline (pre-vaccination), before (CHMI) and 28 days post immunization (CHMI+28 days ) in Tanzanian (n=45) and Equatorial Guinean (n=51) volunteers. Here four volunteers from the whole cohort are displayed as a representation. The figure depicts inter-individual variability in HPgV-1 RNA detection with some individuals negative or positive at one, two or all three measured time points. Log 10 viral loads are plotted on the y-axis and the time points on the x-axis. Each square plot represents an individual with volunteer identification numbers indicated on top. Each dot corresponds to a single time point connected to the next by a solid line. The horizontal dashed line indicates the threshold value of zero viremia. [file 12985_2021_1500_MOESM4_ESM.png]
