# Supplementary material for: Role of human Pegivirus infections in whole Plasmodium falciparum sporozoite vaccination and controlled human malaria infection in African volunteers
Source: Virol J. 2021 Jan 26;18:28. doi: 10.1186/s12985-021-01500-8 (PMC7837505; doi:10.1186/s12985-021-01500-8)
Supplement: Supplementary file 5 — Additional file 5. Table 1 Sensitivity and standard curve ranges for the 45 cytokines, chemokines and growth factors analysed in this study. The tables shows the 45 cytokines, chemokines and growth factors their sensitivities and standard curve ranges as provided by manufacturer. [file 12985_2021_1500_MOESM5_ESM.docx]

| **Parameter** | **Sensitivity** | **Standard curve range** |
| --- | --- | --- |
| BDNF | 1.02 pg/mL | 2.44-10000 pg/mL |
| EGF | 0.85 pg/mL | 2.93-12000 pg/mL |
| Eotaxin | 1.4 pg/mL | 0.61-2500 pg/mL |
| FGF-2 | 2.47 pg/mL | 4.88-20000 pg/mL |
| GM-CSF | 1.2 pg/mL | 17.09-70000 pg/mL |
| GRO-alpha | 2.8 pg/mL | 2.44-10000 pg/mL |
| HGF | 3.10 pg/mL | 8.79-36000 pg/mL |
| IFN alpha | 0.2 pg/mL | 0.61-2500 pg/mL |
| IFN gamma | 0.2 pg/mL | 12.21-50000 pg/mL |
| IL-1 RA | 17.8 pg/mL | 34.18-140000 pg/mL |
| IL 1 alpha | 0.1 pg/mL | 0.61-2500 pg/mL |
| IL beta | 0.2 pg/mL | 2.44-10000 pg/mL |
| IL-2 | 0.8 pg/mL | 4.88-20000 pg/mL |
| IL-4 | 1.5 pg/mL | 12.21-50000 pg/mL |
| IL-5 | 0.3 pg/mL | 7.32-30000 pg/mL |
| IL-6 | 0.4 pg/mL | 9.77-40000 pg/mL |
| IL-7 | 0.2 pg/mL | 0.61-2500 pg/mL |
| IL-8 | 1.2 pg/mL | 2.44-10000 pg/mL |
| IL-9 | 0.5 pg/mL | 9.77-40000 pg/mL |
| IL-10 | 0.1 pg/mL | 2.44-10000 pg/mL |
| IL-12p70 | 0.04 pg/mL | 6.84-28000 pg/mL |
| IL-13 | 0.1 pg/mL | 2.44-10000 pg/mL |
| IL-15 | 1.1 pg/mL | 3.05-12500 pg/mL |
| IL-17A | 0.1 pg/mL | 2.44-10000 pg/mL |
| IL-18 | 0.4 pg/mL | 9.77-40000 pg/mL |
| IL-21 | 0.6 pg/mL | 9.77-40000 pg/mL |
| IL-22 | 8.2 pg/mL | 31.74-130000 pg/mL |
| IL-23 | 0.9 pg/mL | 14.65-60000 pg/mL |
| IL-27 | 5.1 pg/mL | 24.41-100000 pg/mL |
| IL-31 | 3.3 pg/mL | 19.53-80000 pg/mL |
| IP-10 | 0.3 pg/mL | 1.95-8000 pg/mL |
| LIF | 0.83 pg/mL | 6.10-25000 pg/mL |
| MCP-1 | 0.6 pg/mL | 1.22-5000 pg/mL |
| MIP- 1 alpha | 1.1 pg/mL | 2.14-8750 pg/mL |
| MIP -1 beta | 4.7 pg/mL | 6.10-25000 pg/mL |
| NGF beta | 6.19 pg/mL | 7.32-30000 pg/mL |
| RANTES | 0.2 pg/mL | 0.61-2500 pg/mL |
| PDGF-BB | 6.01 pg/mL | 7.32-30000 pg/mL |
| PIGF-1 | 0.23 pg/mL | 1.22-5000 pg/mL |
| SCF | 0.57 pg/mL | 1.59-6500 pg/mL |
| SDF-1alpha | 20.5 pg/mL | 17.09-70000 pg/mL |
| TNF alpha | 0.4 pg/mL | 8.54-35000 pg/mL |
| TNF beta | 1.6 pg/mL | 6.10-25000 pg/mL |
| VEGF-A | 1.73 pg/mL | 5.86-24000 pg/mL |
| VEGF-D | 0.88 pg/mL | 12.21-50000 pg/mL |
